# Toothed whale and shark depredation indicators: A case study from the Reunion Island and Seychelles pelagic longline fisheries

**DOI:** 10.1371/journal.pone.0202037

**Published:** 2018-08-10

**Authors:** Njaratiana Rabearisoa, Philippe S. Sabarros, Evgeny V. Romanov, Vincent Lucas, Pascal Bach

**Affiliations:** 1 Marine Biodiversity, Exploitation, and Conservation (MARBEC), Institut de Recherche pour le Développement, Université de Montpellier, Centre National de Recherche Scientifique, Institut Français de Recherche pour l’Exploitation de la Mer, Saint-Denis, Ile de La Réunion, France; 2 Marine Biodiversity, Exploitation, and Conservation (MARBEC), Institut de Recherche pour le Développement, Université de Montpellier, Centre National de Recherche Scientifique, Institut Français de Recherche pour l’Exploitation de la Mer, Sète, France; 3 Centre technique d’Appui à la Pêche RéUNionnaise (CAP RUN), Le Port, Ile de La Réunion, France; 4 Seychelles Fishing Authority, Victoria, Mahé, Seychelles; Department of Agriculture and Water Resources, AUSTRALIA

## Abstract

Depredation in marine ecosystems is defined as the damage or removal of fish or bait from fishing gear by predators. Depredation raises concerns about the conservation of species involved, fisheries yield and profitability, and reference points based on stock assessment of depredated species. Therefore, the development of accurate indicators to assess the impact of depredation is needed. Both the Reunion Island and the Seychelles archipelago pelagic longline fisheries targeting swordfish (*Xiphias gladius*) and tuna (*Thunnus* spp.) are affected by depredation from toothed whales and pelagic sharks. In this study, we used fishery data collected between 2004 and 2015 to propose depredation indicators and to assess depredation levels in both fisheries. For both fisheries, the interaction rate (depredation occurrence) was significantly higher for shark compared to toothed whale depredation. However, when depredation occurred, toothed whale depredation impact was significantly higher than shark depredation impact, with higher depredation per unit effort (number of fish depredated per 1000 hooks) and damage rate (proportion of fish depredated per depredated set). The gross depredation rate in the Seychelles was 18.3%. A slight increase of the gross depredation rate was observed for the Reunion Island longline fleet from 2011 (4.1% in 2007–2010 and 4.4% in 2011–2015). Economic losses due to depredation were estimated by using these indicators and published official statistics. A loss of 0.09 EUR/hook due to depredation was estimated for the Reunion Island longline fleet, and 0.86 EUR/hook for the Seychelles. These results suggest a southward decreasing toothed whale and shark depredation gradient in the southwest Indian Ocean. Seychelles depredation levels are among the highest observed in the world revealing this area as a “hotspot” of interaction between pelagic longline fisheries and toothed whales. This study also highlights the need for a set of depredation indicators to allow for a global comparison of depredation rates among various fishing grounds worldwide.

## Introduction

The exponential growth of the world's population is putting ever more pressure on food supply at the global scale [1], resulting in increased and more widespread fishing effort [2,3]. Large pelagic fishes like tuna, targeted by fisheries are feeding on the same prey as non-targeted predators resulting in an overlap of their foraging grounds. Interactions with fisheries and human activities represent one of the most significant threats to marine predator populations worldwide [4–6]. These interactions are often classified as biological or operational [7]. Biological interactions are indirect and involve competition between fisheries and marine predators for the same resources [8]. Operational interactions are direct and include (i) intentional and accidental captures, or entanglement of marine mammals and sharks in fishing gears (bycatch) [9–12], and (ii) damage to fishing gear and to captured fish or bait by predators, mostly by toothed whales or sharks [13,14].

Operational interactions include depredation, which is defined as “the partial or complete removal of hooked fish or bait from fishing gear” by marine predators such as cetaceans, sharks, birds, squids, teleost fish, crustaceans and other animals, as opposed to “predation” that is defined as the “taking of free swimming fish (or other organisms)” [13,15]. The first report of toothed whale depredation in the literature dates back to 1952 from Japanese longliners targeting tuna in the waters of Palau [16]. To date, no evidence of increased depredation level has been published. However, the global expansion of longline fisheries associated with more accurate and detailed fishery statistics are providing more frequent depredation reports [17]. Depredation is now considered as a global economic and ecologic issue, occurring in various fisheries, especially those making use of pelagic and demersal longlines [13–15].

Depredation results from opportunistic feeding of predatory species and is a common occurrence among cetaceans. Such behaviours might have adverse consequences on the ecology and conservation of cetaceans. For instance, it may induce a diet shift in toothed whales, resulting from easier access to prey, altering their natural foraging behaviour [13,14]. Interactions with longline gears expose predators to higher risks of getting injured or killed from possible hooking or entanglement [18–21]. Depredation may also affect scientific advice for management of harvested fish stocks since the catch per unit effort (CPUE) data series commonly used in stock assessment processes can be biased due to non-reporting of fish depredated in catch statistics [22]. In addition, depredation may lead to increased fishing effort to compensate for lost catch, resulting in extra fishing pressure on exploited stocks, as well as other non-targeted species [23]. Finally, depredation affects the economics of fisheries. In the Crozet Islands Exclusive Economic Zone (EEZ) demersal longline fisheries, a total of 4.8 million EUR worth of Patagonian toothfish (*Dissostichus eleginoides*) was lost due to killer whale (*Orcinus orca*) and sperm whale (*Physeter macrocephalus*) depredation between 2003 and 2008 [24]. In southern Chile, the same species were responsible for the loss of $93,000 USD of Patagonian toothfish in 2002–2003 [25]. Apart from the direct costs, fishers also bear additional costs to compensate for fish loss. Killer whale depredation avoidance measures account for an average loss of $494 USD per day for food and fuel for Alaskan demersal longliners [26]. Similarly, for Japanese tuna fisheries operating in the Pacific Ocean and in the Indian Ocean in 1976, it was assessed that the amount of damaged product due to false killer whale (*Pseudorca crassidens*) and killer whale depredation would be $50 million USD, if a production value of approximately $900 million USD is considered [23].

Depredation by sperm whales and killer whales on demersal longline fisheries are well documented in sub-Antarctic areas [13,27–29]. Conversely, in tropical areas where pelagic longline fisheries mainly operate, description and quantification of depredation by several smaller toothed whales species and by pelagic sharks is less documented [10,14,16,30–35]. The use of accurate indicators to assess the extent of depredation in pelagic longline fisheries is essential, but is dependent on fishery-based data, which are still scarce and not routinely collected. Additionally, there is a lack of coherence between depredation indices used in the different studies investigating this issue [36]. The aim of this study is to assess and quantify the level of depredation by toothed whales and sharks impacting the pelagic longline fisheries operating from the Reunion Island and Seychelles, western Indian Ocean, using several indices standardized across the two fleets.

## Material and methods

### Data

#### Seychelles pelagic longline fishery

**Fig 1:**
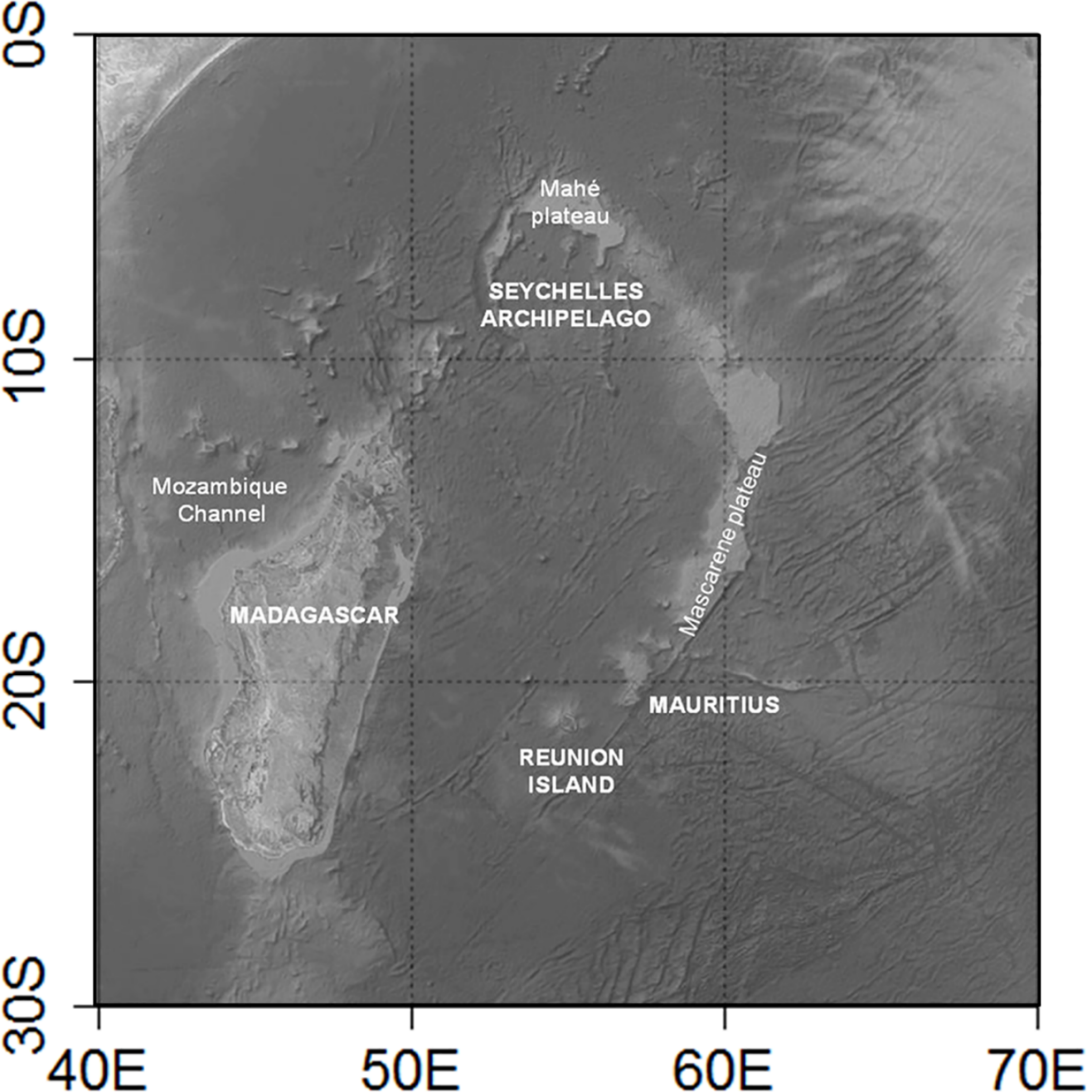
Study area. Map of the southwest Indian Ocean showing general areas from which data were collected (GEBCO bathymetric dataset).

The Seychelles pelagic longline fleet consists of chartered industrial and privately-owned, semi-industrial vessels. This study mainly focused on the locally based, semi-industrial pelagic longline fleet active since 1995 [37] and operating in the Seychelles EEZ (50°E-60°E/0°S-10°S) (Fig 1). The fleet is composed of vessels ranging between 16 m and 23 m in overall length (LOA) which are operated by Seychellois fishers. A data collection program has been implemented by the Seychelles Fishing Authority (SFA) since 1995 to collect catch and effort information from logbooks completed by fishers and landing data from fish processors [38]. Fishing data used in this study were collected between 2004 and 2006. During the study period, the Seychelles semi-industrial monofilament longline fleet consisted of four to six semi-industrial boats targeting swordfish (*Xiphias gladius*) and tuna species (*Thunnus* spp.). These vessels remain at sea for a period of 8 to 12 days and use ice to store their catch [38]. Longlines are usually set at sunset, are left to drift all night and are hauled at sunrise. The mainline can hold from 230 to 900 hooks baited with squid.

Research cruises. Data were collected in the framework of the research program CAPturabilité des grands PElagiques exploités à la Palangre dérivante dans la Zone Economique Exclusive des Seychelles (CAPPES). The aim of the CAPPES program was to study the behaviour of the gear, the habitat of the target species and the efficiency of various baits [39]. During CAPPES surveys, catch and depredation data were reported at the individual level (species, capture status, fate, depredation, and predator group when identification was possible). Fishing strategy was also reported (date, time and location at the beginning and end of each setting and hauling operation). A total of 14 trips representing 70 fishing operations and 30,000 hooks set were analysed in this study. Research trips were mainly carried out in the Seychelles EEZ from 2004 to 2006.

Commercial cruises. Data were extracted from the Fisheries Information and Statistical System (FINSS) developed by the Indian Ocean Tuna Commission (IOTC) to store Seychelles commercial fishery data [40]. Catch and depredation data were reported by fishing operation and by batch of species. Indeed, fish are not individually registered by fishers, and instead, a whole batch of fish is reported in the logbook. Only fish of the same species, caught on the same fishing set are registered together, as a batch. For each fishing set, the start time, end time and location of line setting (and hauling, when possible) were recorded. Hand-written logbooks provided by fishers were used to verify and correct catch and depredation data recorded in the database. The commercial cruises dataset consisted of 92 fishing trips undertaken between 2004 and 2006 in the Seychelles EEZ and represented 710 fishing sets and 445,000 hooks set.

### Reunion Island pelagic longline fishery

The pelagic longline fishery in Reunion Island dates back to 1991 [41]. In 2015, a total of 32 active semi-industrial and industrial vessels, ranging from 8 to 23.9 m in size, were reported. The local pelagic longline fleet mainly targets swordfish, deploying shallow drifting longlines at night, with lightstick-equipped branchlines and baited with squid (or a mix of squid and fish). Fishing vessels remain at sea for a period of 24 hours to 15 days and use ice to store their catch. Longlines are set at depths ranging from 20 to 150 m and hauled at sunrise. The mainline can hold from 300 to 2,000 hooks. Although swordfish is the main target species, some changes in fishing strategy have been undertaken in recent years in order to capture tuna, including increasing the soak time to overlap with tuna feeding activity at the surface [34]. Therefore, bigeye (*Thunnus obesus*), albacore (*Thunnus alalunga*) and yellowfin tuna (*Thunnus albacares*) are also important commercial species.

Catch and fishing tactics data related to the Reunion Island pelagic longline fishery were extracted from the ObServe database [42]. There are three types of data in ObServe: data collected by scientists during research cruises, data collected by observers on commercial fishing cruises, and self-reported data collected by fishers. Fishing operations were undertaken in the Mozambique Channel and off the east coast of Madagascar (40°E-60°E/10°S-30°S) (Fig 1).

Research cruises. Data collected during scientific surveys are more detailed than observer data (supplementary data such as hooking time, hook position on the capture, additional measurement data can also be collected). A total of 10 trips representing 87 fishing operations and 51,200 hooks set were considered. Those trips were undertaken within the framework of several research projects: PROSpection et habitat des grands PElagiques de la ZEE de La Réunion (PROSPER, http://www.iotc.org/news/electronic-tagging-yellowfin-and-bigeye-tuna-prosper-project-phase-2), SouthWest Indian Ocean Fisheries Project (SWIOFP) and Mitigating ADverse Ecological impacts of open ocean fisheries (MADE). Research cruises were undertaken in the Mozambique Channel, Reunion Island and Tromelin EEZs between 2008 and 2012, respectively.

Observer program. The observer program for the Reunion Island-based longline fishery was initiated in March 2007 in the context of the European Data Collection Framework [43]. Data collected by at-sea observers include rigging (line and hook types used, floatlines and branchlines length, detailed gear configuration) and fishing strategy (bait species, date, time and location at the beginning and at the end of each setting and hauling operation). Catch and depredation data were reported at the individual level (species, capture status, fate, position on the longline, depredation and predator group when identification was possible). A total of 74 trips representing 600 fishing operations and 802,000 hooks set were analysed in this study. Observer-monitored trips were mainly carried out in the Reunion Island and Madagascar EEZs between 2007 and 2015.

Self-reporting program. The self-reporting program launched in April 2011 was developed as part of the European Data Collection Framework [44]. In Reunion Island, the fishing activity of longliners below 12 m LOA cannot be monitored by observers. The self-reporting program is used to collect data on the fishing activities of longliners in this category. Captains of longliners willing to take part in the program are required to collect data related to fishing operations (location, date, time and gear configuration), catch composition (total, sold, depredated and discarded catch) and interactions with marine mammals, seabirds and sea turtles. Catch and depredation data are reported per species, by batch and by fishing operation. A total of 341 self-reporting trips representing 1543 fishing operations and 1,944,000 hooks set were analysed. Trips were mainly undertaken in the Reunion Island EEZ and along the east coast of Madagascar from 2011 to 2015.

Data presented in this paper only represent a fraction of the total fishing effort deployed. As a result, the annual coverage rates were also assessed. We used data from official fishing effort statistics [40,45] to estimate the coverage rate as the ratio between the monitored fishing effort (effort with observer coverage and/or effort for which logbooks were available) in number of hooks and the total fishing effort reported for the entire fleet.

### Types of depredation

Depredation is believed to be an important issue in the Seychelles and Reunion Island longline fisheries. From the fisher’s point of view, “globis” or “black fish” (a sub-group defined by fishers and including *Globicephala macrorhynchus* and *Pseudorca crassidens*) are the main species responsible. Both longline fleets also experience catch depredation by large pelagic sharks (*Prionace glauca*, *Carcharinus* and *Isurus* spp, etc.). Depredation of catch by smaller species such as cookie cutter shark (*Isistius brasiliensis*), squids, seabirds, crustaceans or teleost fishes that causes minor damage to target species, and bait depredation by Risso's dolphins (*Grampus griseus*) and bottlenose dolphins (*Tursiops aduncus*), were not considered in this study.

Depredation on catch was quantified using the number of fish damaged and left on the hook by toothed whales and/or sharks. When depredation events were not observed directly, the discrimination between toothed whale and shark attacks was done based on post-mortem analysis of the shape, size and bite pattern on the fish carcass. Sharks generally leave crescent-shaped cuts with clean-cut edges and the overall damage to the fish is often represented by few single bites. Toothed whales leave torn off pieces of flesh, ragged edges of wounds with traces of conical, widely spaced teeth [36,46]. Heavy damage to individual fish is characteristic of toothed whale attacks. They often predate the whole fish leaving only hard parts of the head or up to the position of the hook in the fish body [36,47,48]. However, the discrimination between these depredation types is not always that obvious. Uncertainties regarding the predator group still remain and could not be taken into account in this study, and a bias may arise due to possible misidentifications of predators involved. Only clearly identified depredation type was taken into account in the statistical analyses.

### Catch and depredation indicators

Several catch and depredation indicators were estimated: CPUE, the interaction rate (IR, to assess the frequency of depredation events), the gross depredation rate (GDR, to assess the overall rate of fish lost due to depredation), the depredation per unit effort and the damage rate (DR, to assess depredation impact at the scale of a depredated fishing set), the landing per unit effort (LPUE, to assess depredation impact on landed catch).

IR was computed for all positive sets (on which at least one fish was captured). CPUE, GDR and LPUE were computed for all sets on which at least one commercial fish was captured. Those commercial species include swordfish (as the main target specie) and commercial bycatch species, namely bigeye, albacore and yellowfin tuna, dolphinfish (*Coryphaena hippurus*), black (*Makaira indica*), blue (*Makaira nigricans*) and striped marlin (*Tetrapturus audax*), Indo-Pacific sailfish (*Istiophorus platypterus*) and shortbill spearfish (*Tetrapturus angustirostris*). DPUE and DR were computed for depredated fishing sets on which at least one commercial fish was captured to estimate the impact of depredation at the level of the fishing operation when it occurs. We identified the depredation per unit effort and the damage rate indicators with the symbol * to indicate that they were computed by using depredated sets only.

### Catch indicator

The nominal CPUE, defined as the total number of fish caught (damaged and intact) per 1000 hooks deployed, was assessed per fishing set.

$$\mathrm{CPUE}=\frac{\mathrm{Number}\;\mathrm{of}\;\mathrm{fish}\;\mathrm{caught}}{\mathrm{Number}\;\mathrm{of}\;\mathrm{hooks}\;\mathrm{deployed}}\;x\;1000$$ (1)

### Depredation indicators

The IR is the proportion of longline sets depredated by toothed whales and/or sharks. A fishing operation was considered depredated if at least one fish (either a target species or not) was depredated on the longline. Toothed whale IR (IR_TW_), shark IR (IR_SH_) and double (shark+toothed whale) IR (IR_BOTH_) were assessed by using the whole dataset of fishing operations.

$$\mathrm{IR}=\frac{\mathrm{Number}\;\mathrm{of}\;\mathrm{depredated}\;\mathrm{sets}}{\mathrm{Total}\;\mathrm{number}\;\mathrm{of}\;\mathrm{sets}}$$ (2)

The GDR was defined as the total number of fish depredated by toothed whales and/or sharks divided by the total number of fish caught. Toothed whale GDR (GDR_TW_), shark GDR (GDR_SH_) and double (shark+toothed whale) GDR (GDR_BOTH_) were assessed by using pooled catch data.

$$\mathrm{GDR}=\frac{\mathrm{Number}\;\mathrm{of}\;\mathrm{fish}\;\mathrm{depredated}}{\mathrm{Total}\;\mathrm{number}\;\mathrm{of}\;\mathrm{fish}\;\mathrm{caught}}$$ (3)

The (DPUE*) was defined as the number of fish depredated per 1000 hooks. It was calculated per depredated set. Only fishing sets where at least one commercial fish was depredated by toothed whales and/or sharks were considered to assess DPUE_TW_* and DPUE_SH_* respectively.

$$\mathrm{DPUE}\;*=\frac{\mathrm{Number}\;\mathrm{of}\;\mathrm{fish}\;\mathrm{depredated}}{\mathrm{Number}\;\mathrm{of}\;\mathrm{hooks}\;\mathrm{deployed}}\;x\;1000$$ (4)

The LPUE was defined as the number of fish landed per 1000 hooks, i.e. as the difference between CPUE and DPUE. It was calculated per set. DPUE on non-depredated sets was considered as null, i.e. LPUE_NO_DEP_ (for non-depredated sets) equals CPUE. Only fishing sets where at least one commercial fish was depredated by toothed whales and/or sharks were considered to assess LPUE_TW_* and LPUE_S_* respectively.

LPUE=CPUE−DPUE* (5)

The DR* was defined as the proportion of fish depredated among the total catch at the scale of a fishing operation. DR* was assessed per depredated fishing set. Only fishing sets where at least one commercial fish was depredated by toothed whales and/or sharks were considered to assess DR_TW_* and DR_SH_* respectively.

DR* is the ratio between the number of fish depredated and the number of fish caught.

$$\mathrm{DR}*=\frac{\mathrm{Number}\;\mathrm{of}\;\mathrm{fish}\;\mathrm{depredated}}{\mathrm{Number}\;\mathrm{of}\;\mathrm{fish}\;\mathrm{caught}}$$ (6)

and corresponds to:

$$\mathrm{DR}*=\frac{\mathrm{DPUE}*}{\mathrm{CPUE}}$$ (7)

But DPUE* is also the difference between CPUE and LPUE* (see Eq 5). Therefore, DR* can be considered as the ratio of CPUE not accounted for in catch landings estimations (i.e. reported statistic catch data) when depredation occurs. DPUE* can therefore be presented as:

$$\mathrm{DR}*=\frac{\mathrm{CPUE}‑\mathrm{LPUE}*}{\mathrm{CPUE}}$$ (8)

One overall value of each indicator was also assessed per period, by using pooled data. To do so, two periods were considered: 2004–2010 (Seychelles and Reunion Island data) and 2011–2015 (Reunion Island data, since the beginning of the self-reporting program, leading to the increase of data coverage).

For each period, those indicators were mapped by using (i) five degrees square pooled catch and fishing effort data (for CPUE, GDR and DPUE*) and (ii) the mean of the five degrees square indicator values (for DR*).

Statistical parameters for DR* and DPUE* (minimum, maximum, mean, median, standard deviation, coefficient of variation) were estimated by using a bootstrap procedure [49]. Those statistical parameters were mapped by using a spatial resolution of five degrees square.

Sporadic estimates of shark and/or toothed whale depredation in pelagic longline fisheries can be found in both scientific reports and scientific literature. Despite the fact that those figures were obtained from various methods, we attempted to review the available literature providing some of those depredation rate estimates in S1 Table. The comparison between our results and the literature was analyzed in the Discussion section.

### Assessment of the economic loss due to depredation

A raw assessment of the economic loss (EL) due to shark and toothed whale depredation was done based on the GDR indicator previously calculated. Official landing statistics (by weight) were used for both Reunion Island [45] and Seychelles [40] longline fisheries to assess the theoretical catch without depredation, and the associated fish loss. Then, the catch loss (by weight) due to depredation would be:

$$\mathrm{Catch}\;\mathrm{loss}=\frac{\mathrm{Landings}}{1-\mathrm{GDR}}-\mathrm{Landings}$$ (9)

The average landing price was estimated at 3.3 EUR per kg for the Seychelles (V. Lucas, Chief Fisheries Officer of SFA, pers. comm.) and 4.5 EUR per kg for Reunion Island (H. Chenedé, General Manager of Reunipeche, pers. comm.). The EL due to depredation (in EUR), per fleet and per period (2004–2010 and 2011–2015), was therefore estimated as:

EL=Catchloss*Averagelandingprice (10)

where Catch loss (by weight) was estimated from the weighted landed catch.

To allow comparison between both fleets, the mean economic loss per hook was estimated as the ratio between the estimated economic loss and the total fishing effort reported.

## Results

### Summary of fishing effort, data coverage and capture

#### Seychelles fishing area

**Fig 2:**
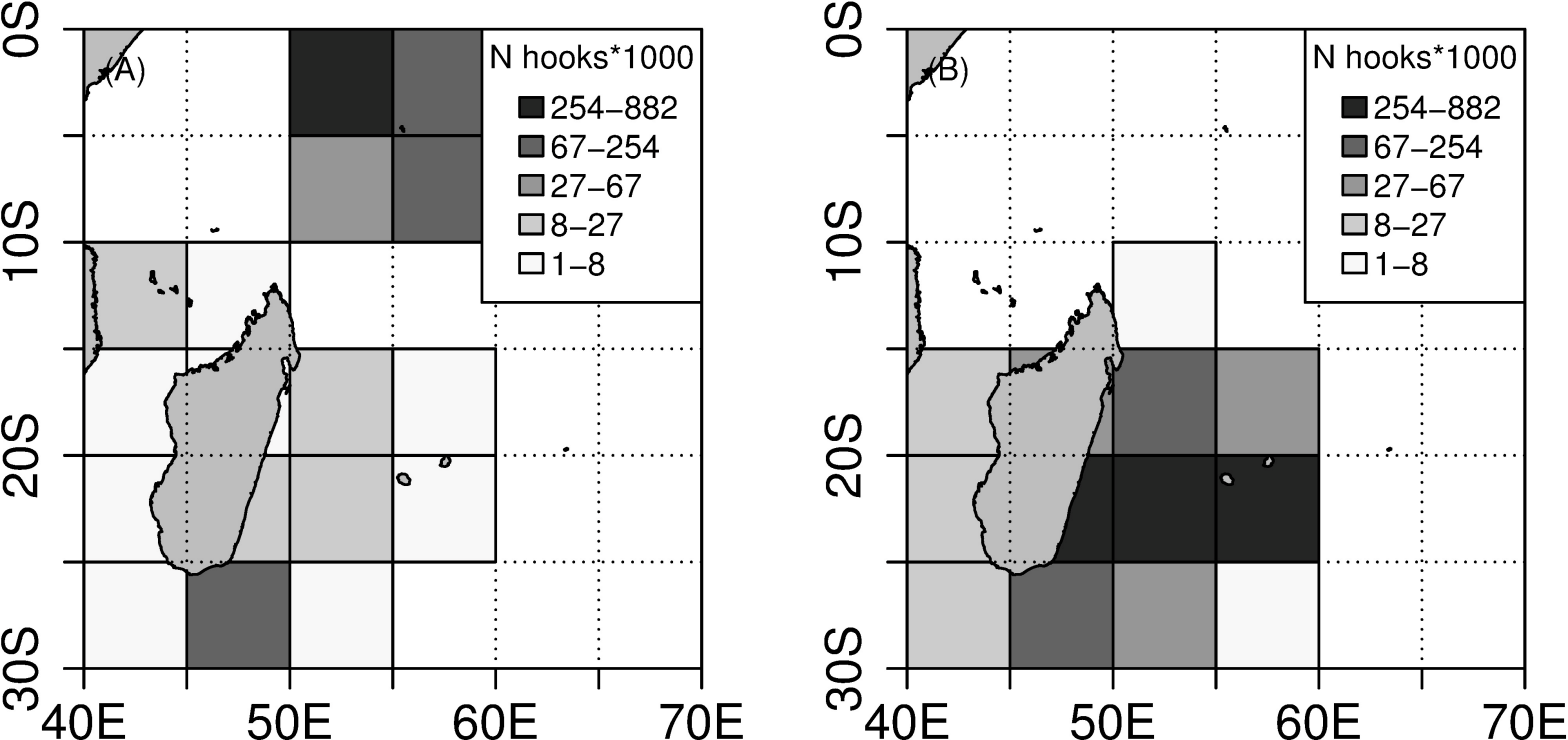
Distribution of fishing effort. (A) Spatial distribution of fishing effort between 2004 and 2010. (B) Spatial distribution of fishing effort between 2011 and 2015.

Fishing operations carried out by the Seychelles small-scale longline fleet were observed within a 300 km radius around the Mahé plateau (Fig 1 and Fig 2). The fishing effort per set averaged 609 hooks and ranged from 176 to 942 hooks. The annual coverage rate ranged between 62 and 98% of the total semi-industrial local longline fleet effort (Table 1). A total of 13,512 fish were caught (61% swordfish, 35% tunas and 4% other species).

**Table 1:** Fishing effort coverage.

	SEYCHELLES			REUNION ISLAND		
Year	Total effort	Data effort	Data coverage (%)	Total effort	Data effort	Data coverage (%)
2004	110000	68795	62.54	2645000	0	0
2005	196000	187298	95.56	3585000	0	0
2006	193000	188751	97.80	3021000	0	0
2007	18631173	0	0.00	4273000	21499	0.5
2008	2459848	0	0.00	3128234	17696	0.57
2009	4337137	0	0.00	3631503	77269	2.13
2010	3640669	0	0.00	3781552	71524	1.89
2011	2885432	0	0.00	3769249	246946	6.55
2012	2971049	0	0.00	3367938	626213	18.59
2013	3493593	0	0.00	4042075	643161	15.91
2014	21366997	0	0.00	3573445	514553	14.4
2015	18694523	0	0.00	3533541	527459	14.93

Yearly total fishing effort (in number of hooks) and data coverage (in %) per fleet. Data effort is the number of hooks for which data was collected. Data coverage is the ratio between data effort and fleet total effort.

Overall, CPUE was estimated at 28.45 fish caught per 1000 hooks when catch and effort data were pooled. Surprisingly, CPUE was lower in the subset of sets not impacted by depredation (22.18 fish per 1000 hooks) compared to those that were depredated by toothed whales and sharks (33.48 and 32.79 fish per 1000 hooks, respectively) (Table 2).

**Table 2:** Summary of depredation indicators.

	Seychelles (2004–2006)				Reunion (2007–2010)				Reunion (2011–2015)			
	No dep.	Dep. TW	Dep. S	Dep. TW&S	No dep.	Dep. TW	Dep. S	Dep. TW&S	No dep.	Dep. TW	Dep. S	Dep. TW&S
**IR (%)**	0	17.1	45	1.3	0	5.4	36.8	8.8	0	9.3	26.2	4.3
**GDR (%)**	0	9.3	8.1	0.9	0	1.7	2.3	0	0	2.3	2	0.4
**Overall CPUE (number of fish caught/1000 hooks)**	22.18	33.48	32.79		26.05	34.27	30.89		16.06	19.58	24.37	
Overall DPUE* (number of fish depredated/1000 hooks)	0	18.56	4.86		0	4.11	1.46		0	3.96	1.36	
Mean DR* (%)	0	56	17		0	21.6	6.1		0	26.8	7.5	
**Overall LPUE (landings/1000 hooks)**	22.18	14.92	27.93		26.05	30.16	29.43		16.06	15.62	23.01	

IR = interaction rate; GDR = gross depredation rate; CPUE = catch per unit effort; DPUE* = depredation per unit effort; DR* = damage rate; LPUE = Landings Per Unit Effort

No dep. = Fishing sets not impacted by depredation; Dep. TW = Fishing sets impacted by toothed whale depredation; Dep. S = Fishing sets impacted by shark depredation; Dep. TW & S = Fishing sets impacted by both depredations

### Reunion Island fishing area

Fishing operations carried out by Reunion Island longline fleet took place in the Mozambique Channel, along the east coast of Madagascar and within the Mascarene plateau (Fig 1 and Fig 2).

For the 2007–2010 period, the annual coverage rate ranged between 0.5 and 1.89% of the total local semi-industrial longline fleet effort (Table 1). The fishing effort per set averaged 1,029 hooks and ranged from 135 to 1,512 hooks. A total of 5,893 fish were caught (32% swordfish, 48% tunas and 20% other species). Overall, CPUE was estimated at 28.08 fish caught per 1000 hooks when catch and effort data were pooled. Similarly to what was observed in the Seychelles, CPUE was lower in the subset of sets not impacted by depredation (26.05 fish per 1000 hooks) compared to those that were depredated by toothed whales and sharks (34.27 and 30.89 fish per 1000 hooks, respectively) (Table 2). These figures are quite similar to those found in the Seychelles fishing area for the 2004–2006 period.

For the 2011–2015 period, the annual coverage rate ranged between 6.55 and 18.59% of the total local semi-industrial longline fleet effort (Table 1). The fishing effort per set averaged 1,277 hooks and ranged from 272 to 1,880 hooks. A total of 47,648 fish were caught (28% swordfish, 43% tunas and 29% other species). Overall CPUE was estimated at 18.41 fish caught per 1000 hooks when catch and effort data were pooled. CPUE was lower in the subset of sets not impacted by depredation (16.06 fish per 1000 hooks) compared to those that were depredated by toothed whales and sharks (19.58 and 24.37 fish per 1000 hooks, respectively) (Table 2).

**Fig 3:**
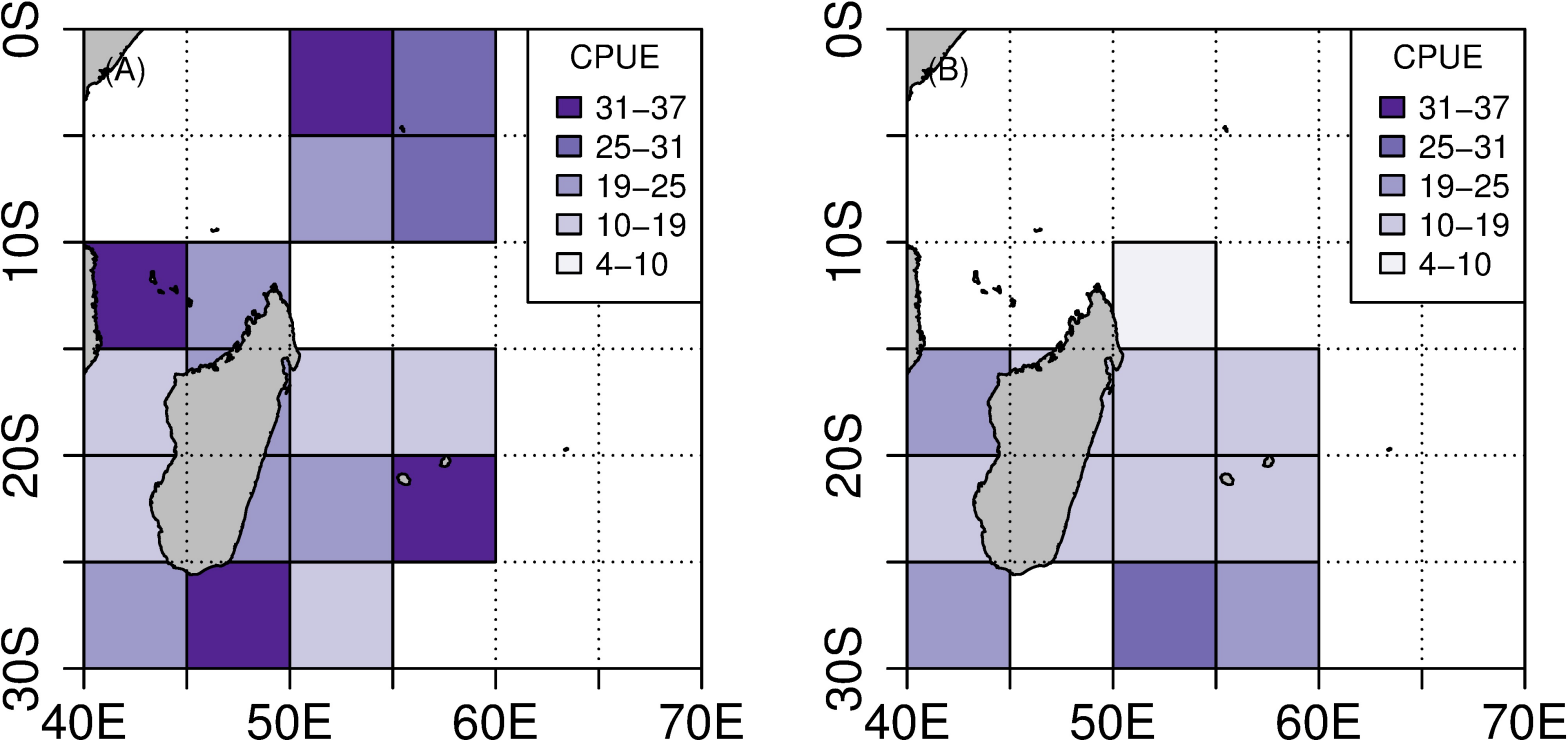
Distribution of CPUE (in number of captured fish per 1000 hooks). (A) CPUE distribution between 2004 and 2010. (B) CPUE distribution between 2011 and 2015.

A significant decrease of the CPUE (H = 60.51, p<0.001) was therefore observed over time (Fig 3). CPUE was higher when shark or toothed whale depredation occurred for the 2007–2010 period. However, a different trend was observed for the 2011–2015 period; CPUE assessed for sets not impacted by depredation was similar to CPUE assessed for sets impacted by toothed whale depredation and lowered over time, while CPUE assessed for sets impacted by shark depredation remained higher.

### Depredation indicators

Depredation indicators were computed to compare the impact of both toothed whale and shark depredation at the scale of the fishery and between fisheries.

### IR

Seychelles fishing area. A total of 494 fishing sets out of 780 were depredated (IR = 63.3%), 133 were depredated by toothed whales (IR_TW_ = 17.1%), 351 were depredated by sharks (IR_SH_ = 45%) and 10 were depredated by both depredators (IR_BOTH_ = 1.3%) (Table 2).

Reunion Island fishing area. A total of 910 fishing sets out of 2230 were depredated (IR = 40.8%), 199 were depredated by toothed whales (IR_TW_ = 8.9%), 605 were depredated by sharks (IR_SH_ = 27.1%) and 106 were depredated by both depredators (IR_BOTH_ = 4.7%).

**Fig 4:**
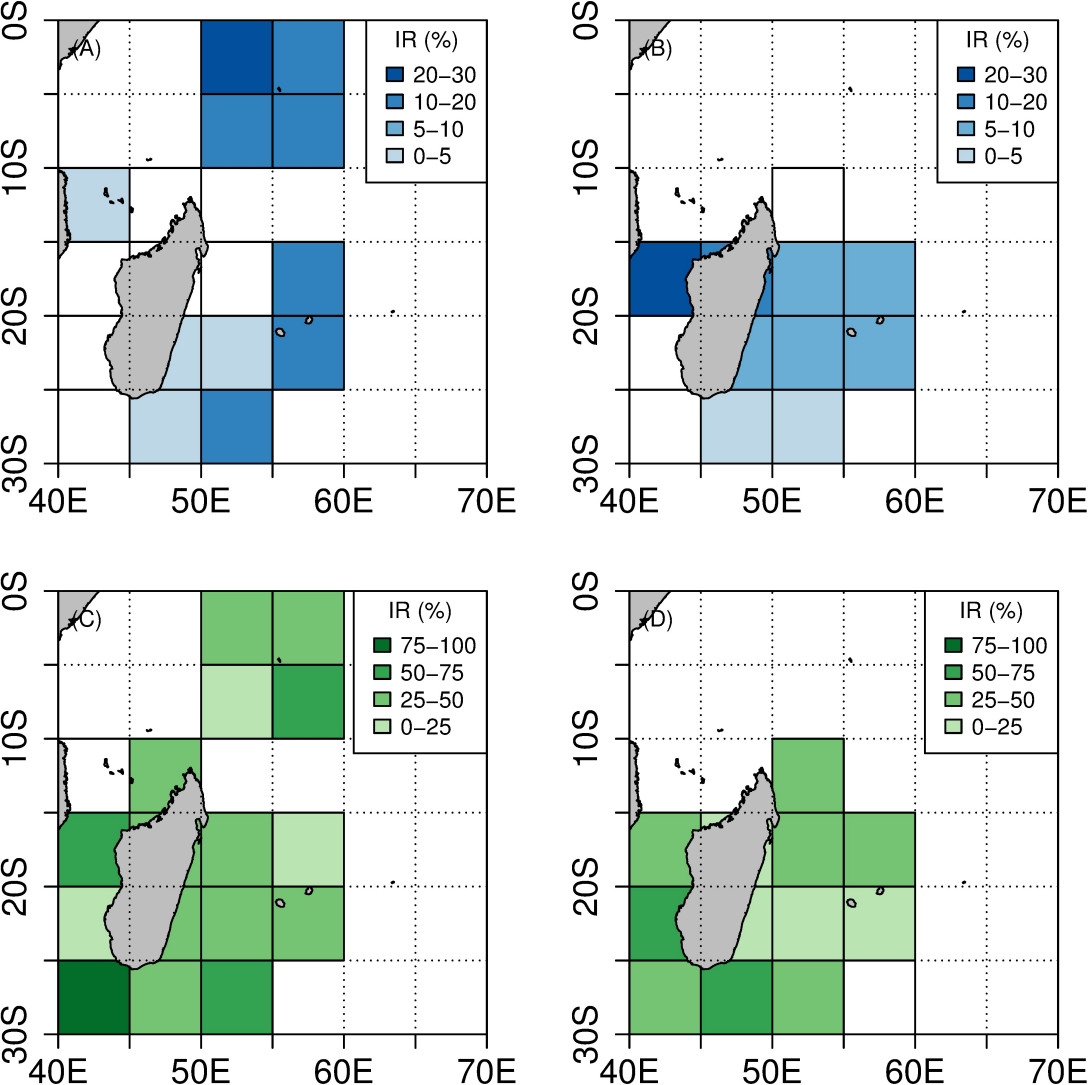
Distribution of IR (in %). IR_TW_ is depicted in blue, IR_SH_ is depicted in green. (A) Distribution of IR_TW_ between 2004 and 2010. (B) Distribution of IR_TW_ between 2011 and 2015. (C) Distribution of IR_SH_ between 2004 and 2010. (D) Distribution of IR_SH_ between 2011 and 2015.

For the 2007–2010 period, the interaction rate was 51% (IR_TW_ = 5.4%, IR_SH_ = 36.8% and IR_BOTH_ = 8.8%). For the 2011–2015 period, the interaction rate was estimated at 39.8% (IR_TW_ = 9.3%, IR_SH_ = 26.2% and IR_BOTH_ = 4.3%). This indicates a 10% decrease of depredated sets with the fishing effort spreading northward and focusing around Reunion Island and alongside the east coast of Madagascar. However, a higher IR_TW_ and a lower IR_SH_ were observed throughout the period (Table 2 and Fig 4).

Due to the lack of data for the second period in the Seychelles, we only considered the 2004–2010 period to compare interaction rates between both areas. Interaction rates between toothed whales and longlines were significantly higher for the Seychelles fishing area than for the Reunion Island fishing area (χ^2^ = 21, p < 0.001). The same result was observed for shark interaction rate (χ^2^ = 9, p = 0.002).

### GDR

Seychelles fishing area. A total of 2,470 individuals out of 13,512 fish (GDR = 18.3%) were predated (GDR_TW_ = 9.3%, GDR_SH_ = 8.1% and GDR_BOTH_ = 0.9%). Swordfish accounted for 70.9% of the total number of depredated fish (Table 2).

Reunion Island fishing area. A total of 2,349 individuals out of 53,541 fish were predated, representing an overall GDR of 4.4% (GDR_TW_ = 2.0%, GDR_SH_ = 2.0% and GDR_BOTH_ = 0.3%), (Table 2). Tuna represented 57.5% of the total number of depredated fish, while 33.6% were swordfish.

For the 2007–2010 period, GDR was estimated at 4.1% (GDR_TW_ = 1.7%, GDR_SH_ = 2.3% and GDR_BOTH_ = 0.0%). For the 2011–2015 period, the overall GDR slightly increased (GDR = 4.4%). However, while GDR_SH_ decreased slightly, GDR_TW_ and GDR_BOTH_ increased. For the 2004–2010 period, the overall GDR was significantly greater for the Seychelles longline fleet than for Reunion Island (χ^2^ = 690, p<0.001). This was also true for GDR_TW_ (χ^2^ = 362, p<0.001) and GDR_SH_ (χ^2^ = 223, p<0.001).

### DPUE*

**Fig 5:**
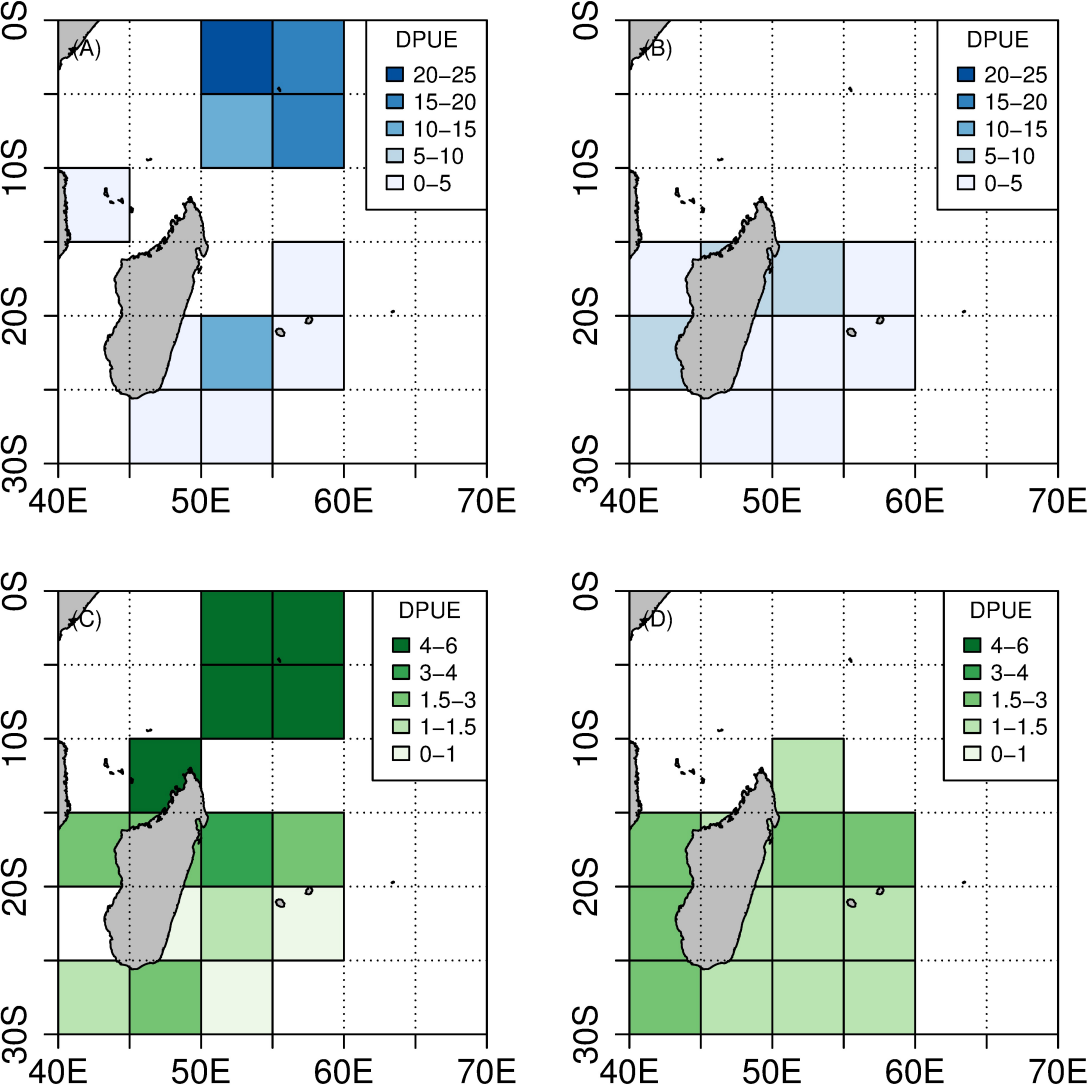
Distribution of DPUE* (in number of depredated fish per 1000 hooks). DPUE_TW_* is depicted in blue, DPUE_SH_* is depicted in green. (A) Distribution of DPUE_TW_* between 2004 and 2010. (B) Distribution of DPUE_TW_* between 2011 and 2015. (C) Distribution of DPUE_SH_* between 2004 and 2010. (D) Distribution of DPUE_SH_* between 2011 and 2015.

Seychelles fishing area. The DPUE_TW_* per set averaged 19.47 depredated fish per 1000 hooks (range = [1.5–76.6], median = 15.7). The DPUE_SH_* per set was lower and averaged 4.95 depredated fish per 1000 hooks (range = [1.1–28.4], median = 3.7). When considering the pooled catch and effort data for the whole period (2004–2006), the overall DPUE_TW_* was equal to 18.56 fish depredated per 1000 hooks, while DPUE_SH_* was more than four times lower (4.80 fish depredated per 1000 hooks) (Table 2 and Fig 5).

Reunion Island fishing area. The DPUE_TW_* per set averaged 4.11 fish depredated per 1000 hooks (range = [0.6–37.0], median = 15.7). The DPUE_SH_* per set was lower and averaged 1.42 fish depredated per 1000 hooks (range = [0.5–7.6], median = 0.9).

When considering the pooled catch and effort data, no trend in DPUE* was detected between 2007–2010 and 2011–2015; this indicator remained relatively constant over time with overall values of 4.11 and 3.96 fish depredated per 1000 hooks, respectively for DPUE_TW_* and 1.46 and 1.36 fish depredated per 1000 hooks respectively for DPUE_SH_* (Table 2 and Fig 5).

A Kruskal-Wallis test showed that DPUE_TW_* was significantly higher than DPUE_SH_* (H = 61.51, p < 0.01 for Reunion Island longline fleet and H = 137.4, p < 0.01 for the Seychelles longline fleet). Moreover, in comparison with fishing operations carried out in Reunion Island, the average DPUE_TW_* and DPUE_SH_* for fishing operations carried out in the Seychelles were significantly higher (H = 135.2, p < 0.01 for DPUE_TW_* and H = 394.25, p < 0.01 for DPUE_SH_*).

This result is supported by the mapped distribution of DPUE_TW_*. The mean and median values of DPUE _TW_* depicted a higher impact of toothed whale depredation in the Seychelles fishing area. The minimum values for DPUE_TW_* were constant over time and between fishing areas. The maximum values for DPUE_TW_* were higher for the Seychelles area, but they also increased over time around Reunion Island. The distribution of the standard deviation and coefficient of variation show high data dispersion over time and space (S1–S6 Figs).

As for DPUE_SH_*, the mean and median values were also higher in the Seychelles area. Over time, they remained constant around Reunion Island. The minimum, maximum, standard deviation and coefficient of variation values were also found to be higher in Seychelles. However, the minimum values decreased while the maximum ones depicted an increasing trend over time around Reunion Island (S1–S6 Figs).

### DR*

**Fig 6:**
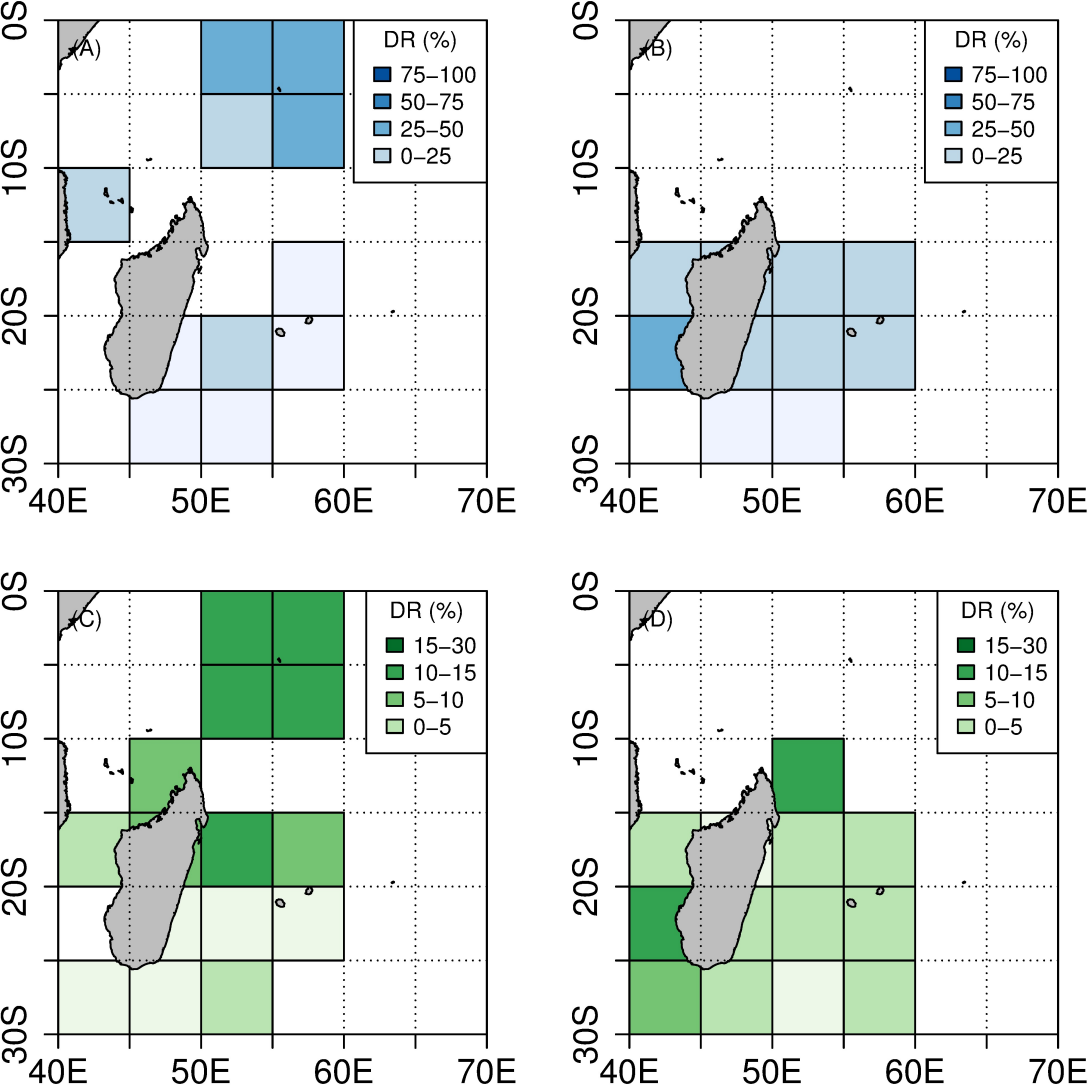
Distribution of DR* (in %). DR_TW_* is depicted in blue, DR_SH_* is depicted in green. (A) Distribution of DR_TW_* between 2004 and 2010. (B) Distribution of DR_TW_* between 2011 and 2015. (C) Distribution of DR_SH_* between 2004 and 2010. (D) Distribution of DR_SH_* between 2011 and 2015.

Seychelles fishing area. When toothed whale depredation occurred, DR_TW_* per set averaged 56% (range = [10–100], median = 53%). This indicates that a mean value of 56% of catch was depredated by toothed whales when interaction with those predators occurred. In contrast, DR_SH_* per set was lower in subsets of sets depredated by sharks and averaged 17% (range = [10–100], median = 14%). DR_TW_* was significantly higher than DR_SH_* (H = 180, p < 0.01). This also indicates that at the scale of a depredated set, the ratio of catch not reported in fisheries statistics was on average three times higher when toothed whale depredation occurred (Table 2 and Fig 6).

Reunion Island fishing area. When toothed whale depredation occurred, DR_TW_* per set averaged 26% (range = [10–100], median = 14%). When shark depredation occurred, DR_SH_* per set averaged 7.3% (range = [10–53], median = 5%). This indicates that a mean value of 26% of catch was unreported in landing statistics when toothed whale depredation occurred, and 7.3% in the case of shark depredation. DR_TW_* was therefore significantly higher than DR_SH_* (H = 91.7, p < 0.01).

A noticeable increase of DR_TW_* was detected between 2007–2010 and 2011–2015. DR_TW_* averaged 21.6 and 26.8% per set for the respective periods, while DR_SH_* remained constant and averaged 6.1 and 7.5% per set, respectively. This indicates that the ratio of fish depredated per set by toothed whales increased to a greater or lesser degree over time, altogether with a decrease of the overall CPUE (Table 2 and Fig 6).

A Kruskal-Wallis test showed that in comparison with fishing operations carried out in the Seychelles EEZ, the average DR_TW_* and DR_SH_* assessed for the Reunion Island fishing area were significantly lower (H = 79.22, p < 0.01 for DR_TW_* and H = 253.83, p < 0.01 for DR_SH_*). It is worth noting that the lowest values of DR_TW_* were observed for fishing operations carried out in the southern part of the Madagascar EEZ before 2011, while DR_SH_* spread rather homogeneously all over the Reunion Island fishing area (Fig 6).

The mean, median, minimum and maximum values of DR_TW_* were found to be higher in the Seychelles waters, but they were also found to increase over time around Reunion Island. On the contrary, standard deviation and coefficient of variation values were lower around the Seychelles (S7–S12 Figs). As for DR_SH_*, similar results were observed when comparing the Seychelles and Reunion Island fishing areas: higher mean and median values were found around the Seychelles. However, those statistics remained constant over time for the Reunion Island. Minimum values were found to be higher around the Reunion Island, but decreased over time. On the contrary, maximum values were higher around the Reunion Island and increased over time. The standard deviation was high around the Seychelles but increased over time around the Reunion Island. The coefficient of variation was high in both areas and remained constant over time (S7–S12 Figs).

### LPUE

For the Seychelles longline fleet, compared with non-depredated sets (LPUE_NO_DEP_ = 22.18 landed fish per 1000 hooks), LPUE_TW_* was substantially lower (LPUE_TW_* = 14.92) while LPUE_SH_* was higher (LPUE_SH_* = 27.93). As for the Reunion Island longline fleet, both LPUE_TW_* and LPUE_SH_* were higher than LPUE_NO_DEP_ between 2007 and 2010. In contrast, LPUE_TW_* was slightly lower than LPUE_NO_ DEP_ while LPUE_SH_* remained higher during the 2011–2015 period (Table 2).

The impact of toothed whale depredation was important as LPUE significantly dropped, especially for the Seychelles and Reunion Island fleets since 2011. Shark depredation impact on the catch was low when it occurred, and LPUE_SH_* remained higher in comparison with sets not affected by depredation.

### Assessment of the economic loss due to depredation

Seychelles fishing area. For the Seychelles longline fleet, the total catch reported for 2004–2006 including swordfish, tuna, sailfish and marlin reached 581 MT [40]. Indexed on the total fishing effort and based on the fish price per kilo, this was equivalent to 3.83 EUR per hook.

Based on Eq (9), it was then assessed that 130 MT were lost to depredation (60 MT due to toothed whale depredation, 51 MT due to shark depredation and 19 MT due to both predators). This corresponds to an estimated economic loss of 429,000 EUR (or 0.86 EUR per hook) for the whole pelagic longline fleet for the 2004–2006 period. This accounted for approximately 22.5% of the fish landed price per hook (Table 3).

**Table 3:** Landings, weight loss and economic loss per fleet and per period.

Fleet	Period	Landings (Mt)	Landings per hook (kg/hook)	Landings per hook (€/hook)	Estimated weight loss (Mt)				Estimated economic loss (k€)				Estimated economic loss per hook (€/hook)				Ratio of depredation (%)
					Total	Dep. TW	Dep. S	Dep. TW&S	Total	Dep. TW	Dep. S	Dep. TW&S	Total	Dep. TW	Dep. S	Dep. TW&S	
Seychelles	2004–2010	581	1.16	3.83	130	60	51	19	429	144	122.4	45.6	0.86	0.4	0.34	<0.01	22.5
Reunion	2007–2010	6982	0.47	2.11	299	121	164	14	1345.5	544.5	738	63	0.09	0.04	0.05	<0.01	4.3
Reunion	2011–2015	7322	0.4	1.8	337	157	149	31	1516.5	706.5	670.5	139.5	0.08	0.04	0.04	<0.01	4.4

Dep. TW = Fishing sets impacted by toothed whale depredation; Dep. S = Fishing sets impacted by shark depredation; Dep. TW & S = Fishing sets impacted by both depredations

The ratio of depredation is the ratio between the total estimated loss per hook (in EUR/hook) and the landings per hook (in EUR/hook).

Reunion Island fishing area. For the whole Reunion Island longline fleet, total catches of 6,982 MT and 7,322 MT were reported for 2007–2010 and 2011–2015, respectively [45]. Indexed on the total fishing effort and based on the fish price per kilo, this was equivalent to 2.11 and 1.80 EUR per hook. Following the same method, the catch loss due to depredation was estimated at 299 MT (121 MT to toothed whale depredation, 164 MT to shark depredation and 14 MT to both predators) for the 2007–2010 period. This corresponds to an estimated economic loss of 1,345,500 EUR (or 0.09 EUR per hook) for the whole longline fleet. This accounted for approximately 4.3% of the fish landed price per hook (Table 3).

For the 2011–2015 period, 337 MT were lost to depredation (157 MT to toothed whale depredation, 149 MT to shark depredation and 31 MT to both predators). This corresponds to an estimated economic loss of 1,516,500 EUR (or 0.08 EUR per hook) for the whole longline fleet. This accounted for approximately 4.4% of the fish landed price per hook (Table 3).

## Discussion

### Comparative impact of toothed whale and shark depredation

Shark and toothed whale depredation impacting the Seychelles and Reunion Island pelagic longline fisheries displayed a similar pattern. Overall, shark depredation events were more frequent than toothed whale ones. However, toothed whales were responsible for more damaged fish on the longline during depredation events. Shark and toothed whale depredation rates differed because toothed whale attacks are generally done by several individuals. Sharks seem to attack fish randomly (and damage only a few of individuals on the longline) whereas toothed whale groups seem to depredate longlines in a methodical way, taking fish one after the other along the line [19,35]. These collective feeding events lead to high toothed whale depredation rates at the level of the set.

The first study undertaken in the Indian Ocean reported an average depredation rate by killer whales of 55% [35]. In Brazil, killer whale depredation events were less frequent than shark depredation events and killer whales took more fish on the line compared to sharks [30,50]. Depredation data from a Soviet historical database assessed that damage per set by toothed whales was twice as high as shark damage in the Indian Ocean [36]. In the waters off Brazil and the Azores archipelago, even if the proportion of sets depredated by toothed whales was low compared to sharks, catch lost to toothed whales was higher [51]. These results are consistent with ours; although toothed whale depredation was less frequent than shark depredation, more fish were damaged on the longline when toothed whale depredation occurred.

### Depredation, unreported CPUE and study limits

Depredation may have substantial impacts on both stock assessments and CPUE analyses. It may in turn affect scientific advice for management of exploited stocks impacted by depredation. Indeed, depredated fish are not reported in landed catch, leading to an underestimation of reported CPUEs and inducing biases in quantitative assessments of available resources when providing management advice [15]. In the Seychelles for instance, 18.3% of the total catch were depredated by sharks and toothed whales. Thus, depredation may have induced an underestimation of 18.3% of the total reported catch for the Seychelles semi-industrial longline fleet between 2004 and 2006. For the Reunion Island semi-industrial longline fleet, this bias reached 4.1% and 4.4% for the 2007–2010 and 2011–2015 periods, respectively. A correction factor should therefore be applied when performing fish stock assessments. However, this correction factor should exclusively be applied on the species and fishing fleet considered when assessing the indicators. In the particular case of the Seychelles semi-industrial longline fleet, this correction factor may be biased if applied on the actual fishery data, as it was estimated using 2004–2006 fishery data.

However, that correction factor cannot take into account several uncertainties, since this study estimated depredation that was apparent. Indeed, depredation rates presented here were assessed based on fish remains and did not account for possible unseen depredation. For instance, toothed whale depredation may lead to complete removal of fish on the hook. That non-quantified depredation may result in further underestimation of toothed whale depredation. Furthermore, catch depredated by sharks may still be traded, since damage can be limited. In such cases, shark depredation indicators assessed here likely overestimate the actual depredation impact. Therefore, estimates presented in this study assessed depredation that was apparent, and potential depredation correction factors that could be implemented in stock assessment may be biased.

A bias might also arise from the uncertainty of predator group identification, as discrimination between shark and toothed whale depredation from fish carcasses is not always obvious. Indeed, a possible misidentification might occur when fishers report depredation events. Those biases could not be quantified and taken into account in this study, and may likely lead to an underestimation of the real depredation in pelagic longline fisheries considered. Moreover, depredation by other species (birds, squids) was not systematically reported. However, those species are thought to produce minor damages to the catch.

### Economic loss due to depredation

Very few studies have been undertaken to assess economic costs associated with depredation. This is especially true for pelagic longline fisheries. The present study aimed to provide an initial assessment of the economic loss due to depredation in two local fisheries operating in the southwest Indian Ocean. Based on fisheries statistics, we estimated that around 429,000 EUR were lost to depredation over three years in the Seychelles longline fishery. This may seem low compared to the loss faced by the Reunion Island longline fishery (1,345,500 EUR in 2007–2010 and 1,516,500 EUR in 2011–2015). However, indexed on the total fishing effort of the whole fleet, economic loss per hook was almost ten times higher in the Seychelles (0.86 EUR per hook versus 0.09 EUR per hook). Those losses per hook accounted for substantial proportions of the estimated price of landed fish per hook (22.5% and 4.4% for the Seychelles and Reunion Island fleets, respectively).

However, as discussed above, depredation rates estimated here only accounted for apparent depredation and economic losses are therefore likely underestimated. But this issue also leads to other non-estimated economic losses. These indirect costs include:

- damage to fishing gear, when toothed whales are caught on the line or when they struggle to take a fish from the hook [52]. This may result in a loss of material and time, when the line has to be cut/fixed and the hauling to be interrupted.
- additional working time, bait and fuel expenses when moving to another fishing area to avoid predators, as demonstrated for demersal longline fisheries [24,29]. In long-distance demersal longline fisheries, spatial displacement of fleets from a fishing area where depredation has occurred significantly reduces toothed whale depredation [24]. However, for small pelagic semi-industrial vessels, this strategy may result in high running costs and heavy operational losses. Thus, depredation is likely more detrimental to small-scale fisheries undertaking short fishing trips (lasting from a few days to three weeks). Moreover, small semi-industrial vessels are usually “fresh-fish boats” preserving their catch on ice. If vessel operators extend the duration of the fishing trip to compensate for depredation losses, they may have to discard their catches from earlier operations due to quality deterioration (“high-grading” process). This may lead to an additional fishing pressure on target fish species [15].
- depredation of bait by toothed whales resulting in empty hooks, inducing a reduced efficiency of the fishing gear and therefore a nominal CPUE decrease [53–55]. Indeed, short-finned pilot whales and false killer whales have also been observed depredating bait [19,56–58].

The overall loss undergone by the Reunion Island pelagic longline fleet was relatively low. However, the overall profitability of this fishery relies on a thin margin, due to high running costs and low fish prices. As a result, even minor losses may significantly reduce profits [34].

### Why apply depredation indicators?

Several depredation indicators were applied in this study. This allowed us to assess the impact of depredation at various levels including: in terms of proportion of impacted fishing sets, proportion of depredated catch (globally or on depredated sets), amount of fish lost per 1000 hooks, and proportion of fish lost from the total catch. The review of available literature shows that several measurements of depredation were used in other studies and no standard index of depredation has been implemented so far. Also, the depredation definition can differ, depending on how it was applied. Thus, depredation can be defined and quantified as the percentage of damaged fish (in weight or in number) among the overall catch [35,59,60], the percentage of damaged fish in sets impacted by depredation exclusively [30,35,36], the percentage of sets or trips affected by depredation [50] or the economic loss. However, because the estimates used in those previous studies differed in their definition, no comparison of depredation rates between fisheries and fishing areas is possible. Therefore, at least one standard set of depredation indices must be defined for comparison purposes. This would allow us to address various aspects of the depredation issue including the percentage of depredated fishing operations, the overall proportion of depredated catch and the depredation impact at the scale of a depredated fishing set. An optimal indicator should be informative about the spatiotemporal intensity of depredation, should allow estimation of the impact of depredation on CPUE, provide a correction factor that could be used in stock assessments and allow an assessment of economic losses. In our study, DPUE* appears to include all those criteria, although the optimal indicator should also take into account the cost of access to information. Based on that condition, DPUE* is not easily accessible, since catch, depredation and effort data are not readily available per fishing set. Collecting such detailed information is time consuming and requires the availability of an on-board observer, an electronic monitoring system or the co-operation from voluntary captains.

Since information about the fishing effort or the number of fishing operations or trips is not always available, the index that would be the easiest to implement is GDR. When working on fishery-dependent data (especially if data are provided by fishers), landings data are the most relevant and available information in every fishery. But other indicators can also be considered. For instance, IR could be a relevant index, as it depicts depredation occurrence. If additional and more detailed information is available (such as fishing effort or catch and depredation data per fishing operation), DR* or DPUE* can also be relevant depredation descriptors.

### Geographic comparison of depredation rates

Toothed whale depredation impact is heavier in the Seychelles, in terms of frequency and intensity. This high depredation level may be the result of a high biological richness in this area, gathering more pelagic fish and predators. Comparison of CPUE between the Seychelles and Reunion Island fishing areas is consistent with this assumption, as catch rate is significantly higher in the Seychelles. Furthermore, results from an aerial survey conducted in the west Indian Ocean suggested a higher marine mammal diversity index in the waters around the Seychelles [61]. Larger group size and higher encounter rate of false killer whales and short-finned pilot whales were also observed in that area. Southward, in the fishing areas where the Reunion Island fleet operates, toothed whale populations involved in depredation seem to depredate less frequently and at a lesser level. Depredation occurring in those waters is less of an issue in terms of amount of fish lost, compared to the Seychelles.

For the study period, GDR sustained by the Seychelles fleet reached 18.3%. To date, this is one of the highest GDR reported for tropical waters, where GDR ranged from 0.2 to 15% [16,32,35,36,50,51,59,60,62–69] (S1 Table). For the Reunion Island fleet, GDR was within the range of values observed in other areas. Our results are consistent with previous work undertaken in our study area. For instance, in the Seychelles, it was assessed that the yearly GDR ranged from 14 to 27% [62]. For the Reunion Island pelagic longline fleet, a previous study reported that GDR_TW_ and GDR_SH_ reached 4% and 3%, respectively [32]. In a recent study carried out in the southern Indian Ocean, GDR was similar to that estimated for the Reunion Island fleet in the current study (3%) [59].

IR assessed for the Seychelles and Reunion Island pelagic longline fleets were clearly greater than those assessed in other regions, where shark interaction rates ranged from 20 to 25.6% [36,50], while toothed whale interaction rates ranged from 1.6 to 6.2% [36,50,51,60,70,71]. When considering depredated sets only, the Seychelles indicators were consistent with those found in the literature: 11 to 21% and 18 to 55% of the catch are lost to shark and toothed whale depredation, respectively [30,35,36,68,72]. For the Reunion Island fleet, the indicators were slightly lower than the ones found in the literature for shark depredation, but within the range of reported values for toothed whale depredation (S1 Table).

The south western Indian Ocean appears to be a fishing area that is frequently affected by both toothed whale and shark depredation. Indeed, based on the comparative values of depredation indicators calculated for the Seychelles and Reunion Island pelagic longline fisheries, the Seychelles appears to be a depredation hotspot. More precisely, there might be a southward decreasing toothed whale and shark depredation gradient in the southwest Indian Ocean. However, although toothed whale depredation indicators were found to be lower in Reunion Island fishing areas than in Seychelles, toothed whale depredation significantly reduced the amount of landed fish and is therefore likely to have a high impact on the fishery’s profits.

## Conclusion

This study showed that (i) interactions with pelagic longlines involving sharks are more frequent than the ones involving toothed whales; (ii) the median loss per set is higher when toothed whale depredation occurs; (iii) depredation mainly occurs in areas of high resource abundance; (iv) there is a southward decreasing depredation gradient in the western Indian Ocean; (v) the Seychelles is likely a depredation hotspot at the scale of both the Indian Ocean and the world oceans; (vi) in the south western Indian Ocean, depredation would lead to an underestimation of 18.3% and around 4% of CPUE data considered in stock assessment (for the Seychelles and for Reunion Island fleets, respectively); vii) the economic loss was estimated at 0.09 and 0.86 EUR per hook (accounting for 4.4% and 22.5% of the landed price per hook) for the Seychelles and Reunion Island fisheries, respectively.

Given the negative impacts of depredation on fishers, predators and target fish, it is crucial to monitor this phenomenon. However, this is a challenging issue, given the lack of knowledge about the ecology and migration patterns of the species involved. Depredation monitoring should involve both scientists and fishers, and include the development of standard data sheets for data collection, the use of standard depredation indices and appropriate quantification methods. Despite the impact of depredation on pelagic longline fisheries being poorly understood, especially in the western Indian Ocean, this study provides valuable insights that can be applied in future studies to improve our understanding of this issue [34]. A future study of the economic impact of depredation will be especially important, and should include estimations of the direct costs, as well as indirect costs (e.g. salary, fuel consumption etc) incurred by fisheries.

This study highlighted the heavy impacts of shark and toothed whale depredation on pelagic longline fleets. Many depredation mitigation measures have been tested so far in this fishery, including acoustic devices and physical protection of the catch [17,73–75]. However, this remains challenging due to the learning skills and habituation of predators to new mitigation methods. To meet fishers’ demand to mitigate this issue, we are currently undertaking work to develop a physical depredation mitigation device [55,76,77].

## Supporting information

S1 Table Review of the available literature about depredation impacting pelagic longline fisheries. Target species abbreviations: Billfish (BIL): Swordfish (SWO); Unidentified shark species (SHK); Tuna species (TUN).
Predator species abbreviations: Killer whale (KW); False killer whale (FKW); Short-finned pilot whale (SFPW); Unidentified shark species (SHK); Unidentified toothed whale species (TW).
Depredation calculation method: Interaction rate = depredated fishing sets/total number of fishing sets; Depredation rate = fish damaged / total number of fish caught (including damaged); Depredated sets: depredation rate calculated on depredated sets; All sets: depredation rate calculated on all positive sets (i.e. including non affected sets but with at least one fish caught).
Metric: Depredation rate calculated as the proportion of fish lost in number (Nb); Depredation rate calculated as the proportion of fish lost in weight (W).
* recalculated from swordfish and tuna catch and depredation data.
(XLSX)

S1 Fig Mean DPUE. Mean DPUE in number of depredated fish per 1000 hooks (left: 2004–2010, right: 2011–2015; blue: toothed whale depredation, green: shark depredation).
(TIF)

S2 Fig Median DPUE. Median DPUE in number of depredated fish per 1000 hooks (left: 2004–2010, right: 2011–2015; blue: toothed whale depredation, green: shark depredation).
(TIF)

S3 Fig Minimum DPUE. Minimum DPUE in number of depredated fish per 1000 hooks (left: 2004–2010, right: 2011–2015; blue: toothed whale depredation, green: shark depredation).
(TIF)

S4 Fig Maximum DPUE. Maximum DPUE in number of depredated fish per 1000 hooks (left: 2004–2010, right: 2011–2015; blue: toothed whale depredation, green: shark depredation).
(TIF)

S5 Fig Standard deviation of DPUE. Standard deviation of DPUE in number of depredated fish per 1000 hooks (left: 2004–2010, right: 2011–2015; blue: toothed whale depredation, green: shark depredation).
(TIF)

S6 Fig Coefficient of variation of DPUE. Coefficient of variation of DPUE in number of depredated fish per 1000 hooks (left: 2004–2010, right: 2011–2015; blue: toothed whale depredation, green: shark depredation).
(TIF)

S7 Fig Mean DR. Mean DR in % (left: 2004–2010, right: 2011–2015; blue: toothed whale depredation, green: shark depredation).
(TIF)

S8 Fig Median DR. Median DR in % (left: 2004–2010, right: 2011–2015; blue: toothed whale depredation, green: shark depredation).
(TIF)

S9 Fig Minimum DR. Minimum DR in % (left: 2004–2010, right: 2011–2015; blue: toothed whale depredation, green: shark depredation).
(TIF)

S10 Fig Maximum DR. Maximum DR in % (left: 2004–2010, right: 2011–2015; blue: toothed whale depredation, green: shark depredation).
(TIF)

S11 Fig Standard deviation of DR. Standard deviation of DR in % (left: 2004–2010, right: 2011–2015; blue: toothed whale depredation, green: shark depredation).
(TIF)

S12 Fig Coefficient of variation of DR. Coefficient of variation of DR in % (left: 2004–2010, right: 2011–2015; blue: toothed whale depredation, green: shark depredation).
(TIF)
