# Supplementary figures and images for: Toothed whale and shark depredation indicators: A case study from the Reunion Island and Seychelles pelagic longline fisheries
Source: PLoS One. 2018 Aug 10;13(8):e0202037. doi: 10.1371/journal.pone.0202037 (PMC6086455; doi:10.1371/journal.pone.0202037)

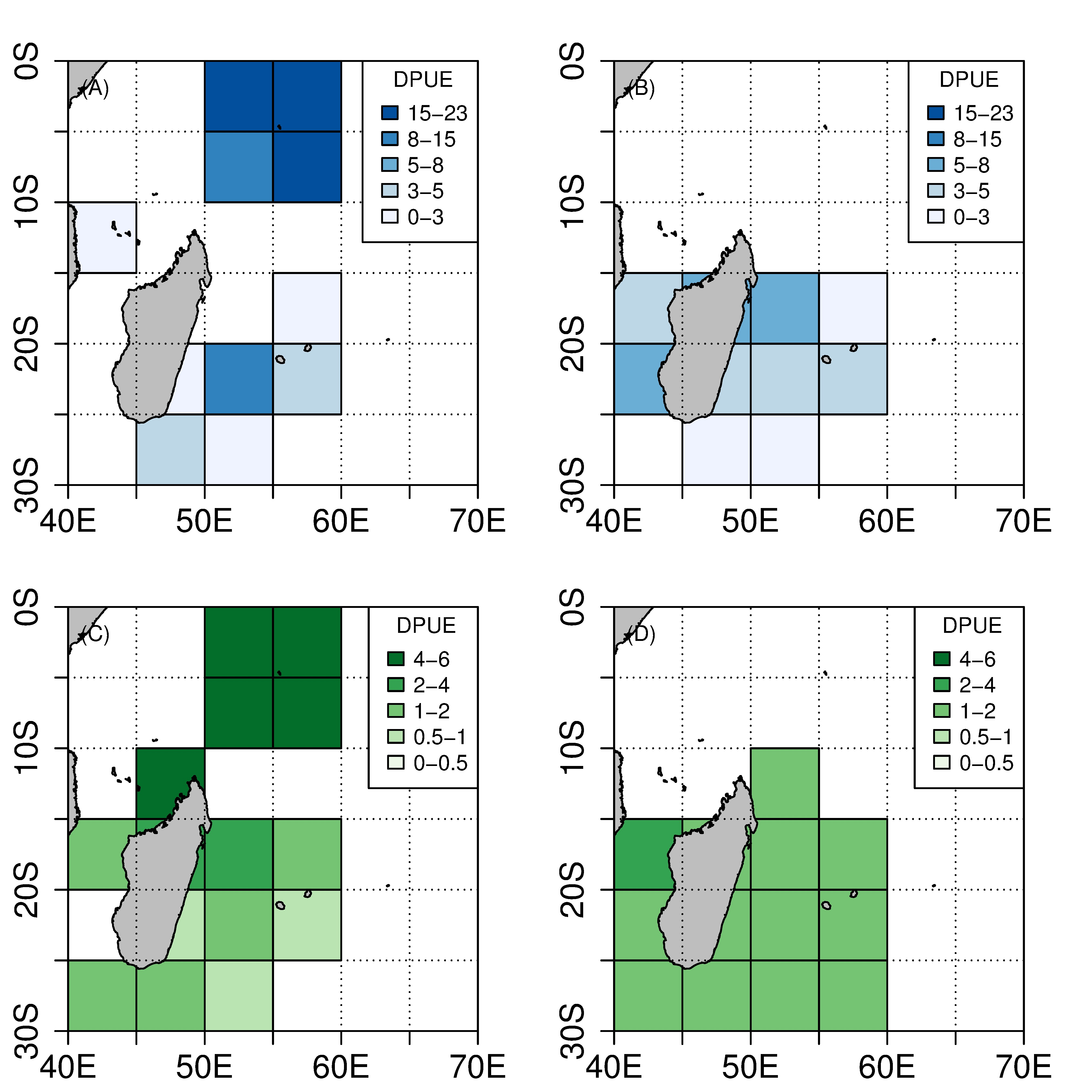

Supplement: S1 Fig — Mean DPUE in number of depredated fish per 1000 hooks (left: 2004–2010, right: 2011–2015; blue: toothed whale depredation, green: shark depredation). (TIF) [file pone.0202037.s002.tif]

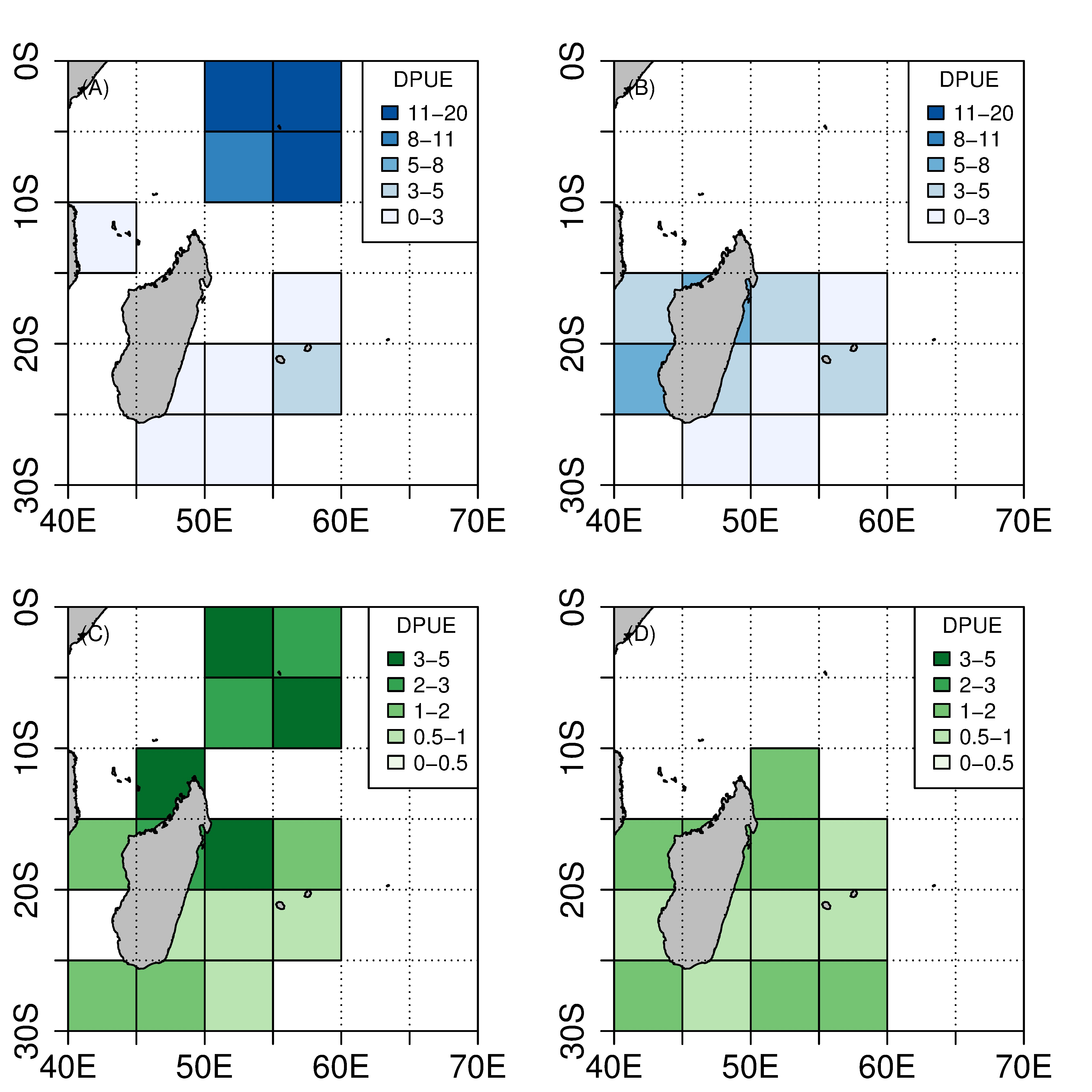

Supplement: S2 Fig — Median DPUE in number of depredated fish per 1000 hooks (left: 2004–2010, right: 2011–2015; blue: toothed whale depredation, green: shark depredation). (TIF) [file pone.0202037.s003.tif]

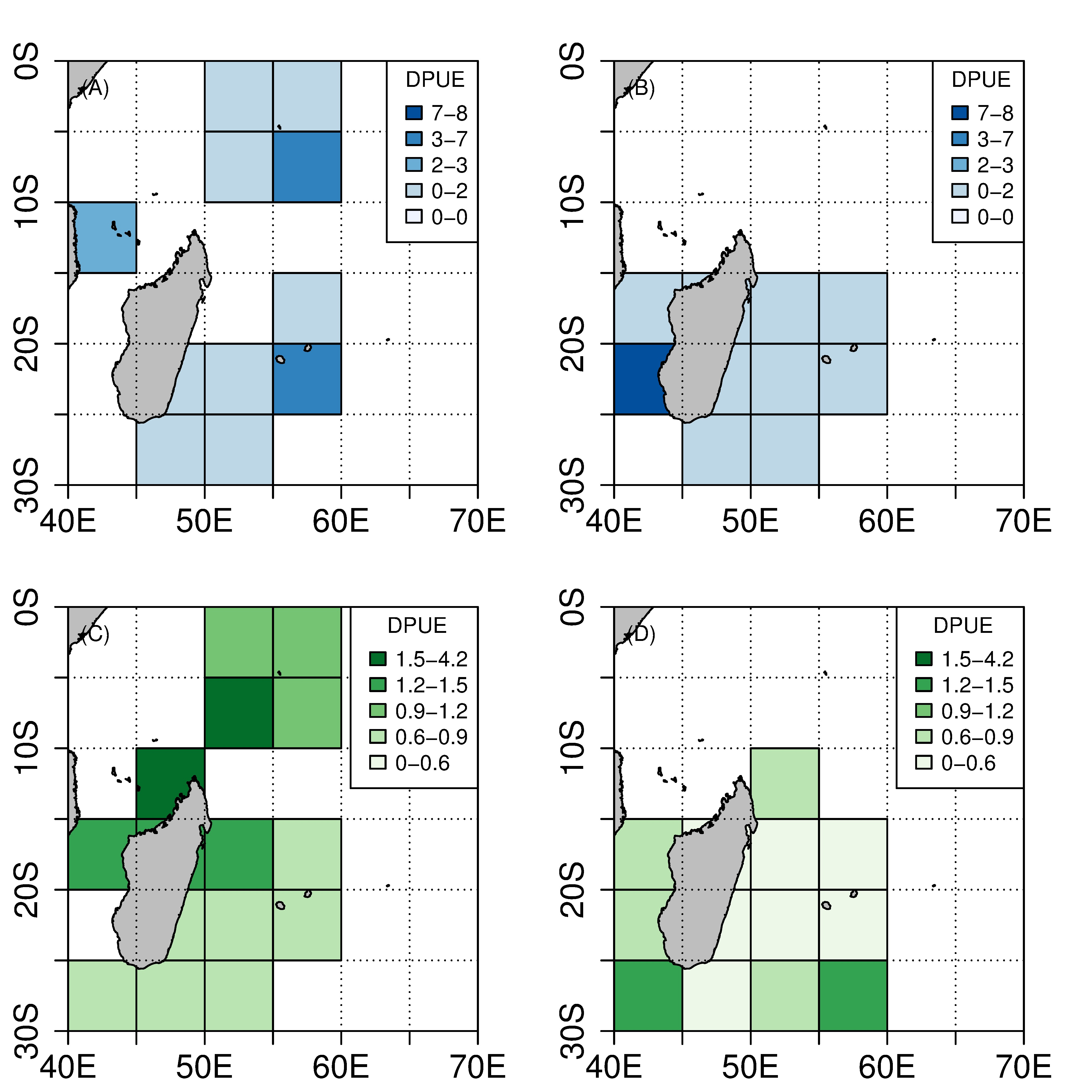

Supplement: S3 Fig — Minimum DPUE in number of depredated fish per 1000 hooks (left: 2004–2010, right: 2011–2015; blue: toothed whale depredation, green: shark depredation). (TIF) [file pone.0202037.s004.tif]

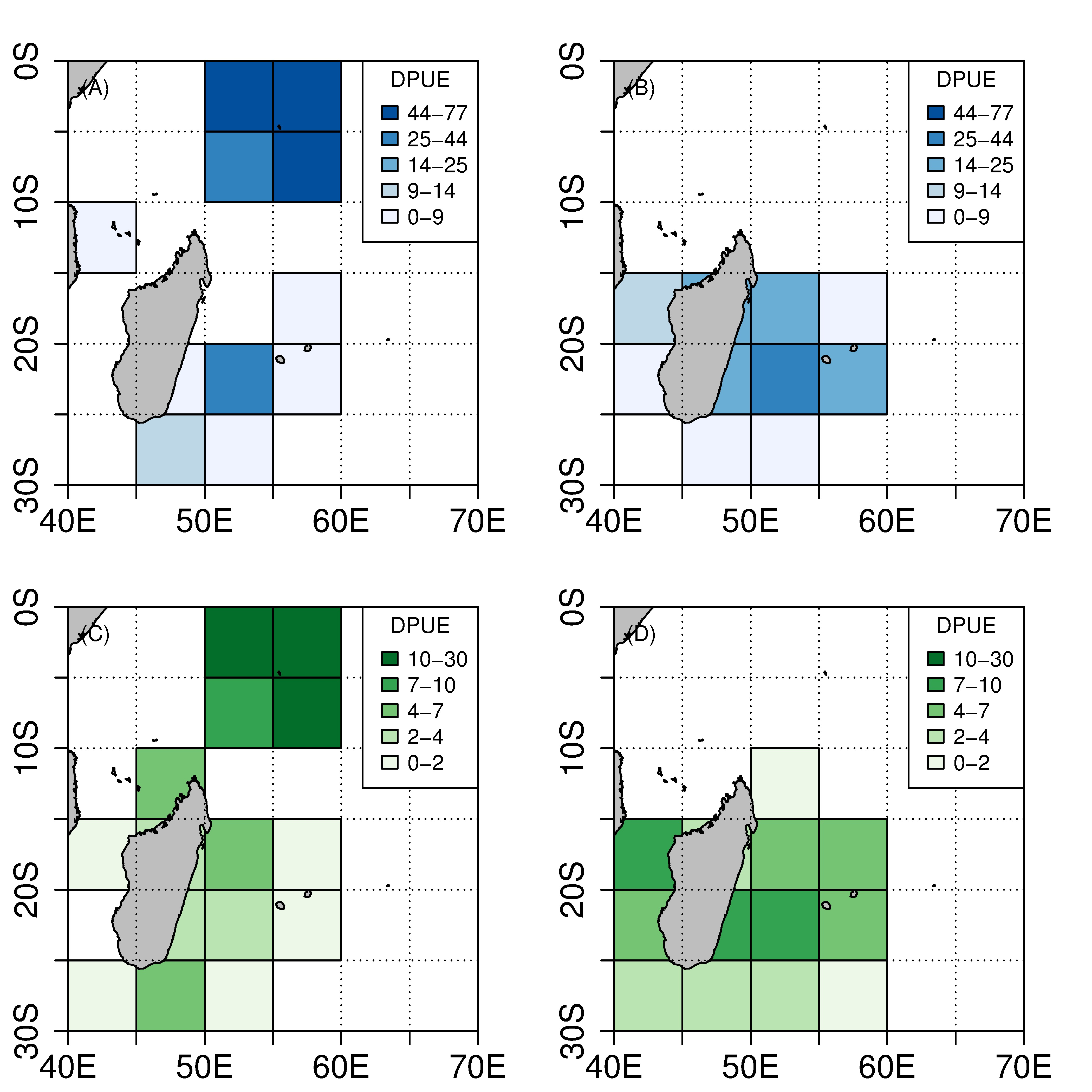

Supplement: S4 Fig — Maximum DPUE in number of depredated fish per 1000 hooks (left: 2004–2010, right: 2011–2015; blue: toothed whale depredation, green: shark depredation). (TIF) [file pone.0202037.s005.tif]

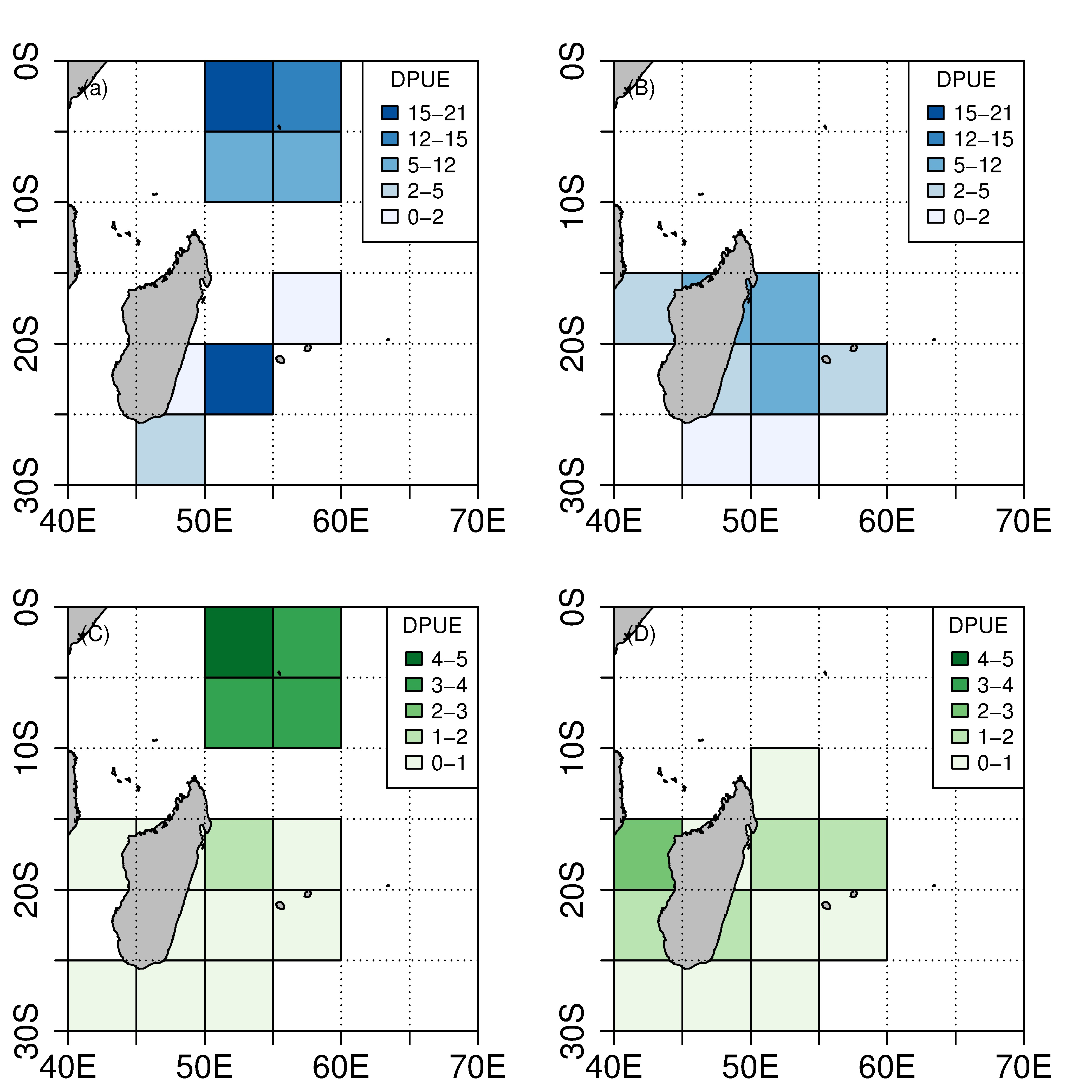

Supplement: S5 Fig — Standard deviation of DPUE in number of depredated fish per 1000 hooks (left: 2004–2010, right: 2011–2015; blue: toothed whale depredation, green: shark depredation). (TIF) [file pone.0202037.s006.tif]

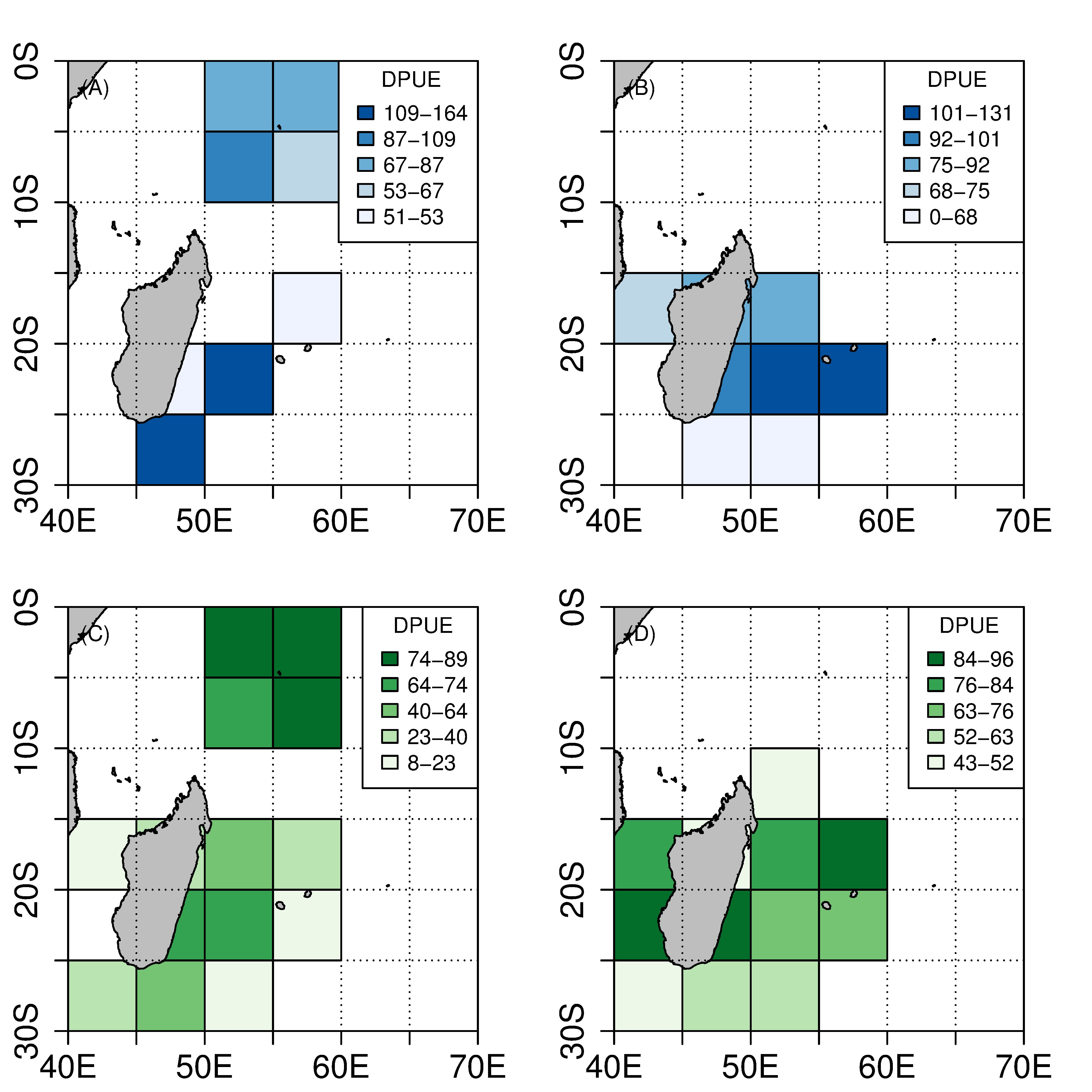

Supplement: S6 Fig — Coefficient of variation of DPUE in number of depredated fish per 1000 hooks (left: 2004–2010, right: 2011–2015; blue: toothed whale depredation, green: shark depredation). (TIF) [file pone.0202037.s007.tif]

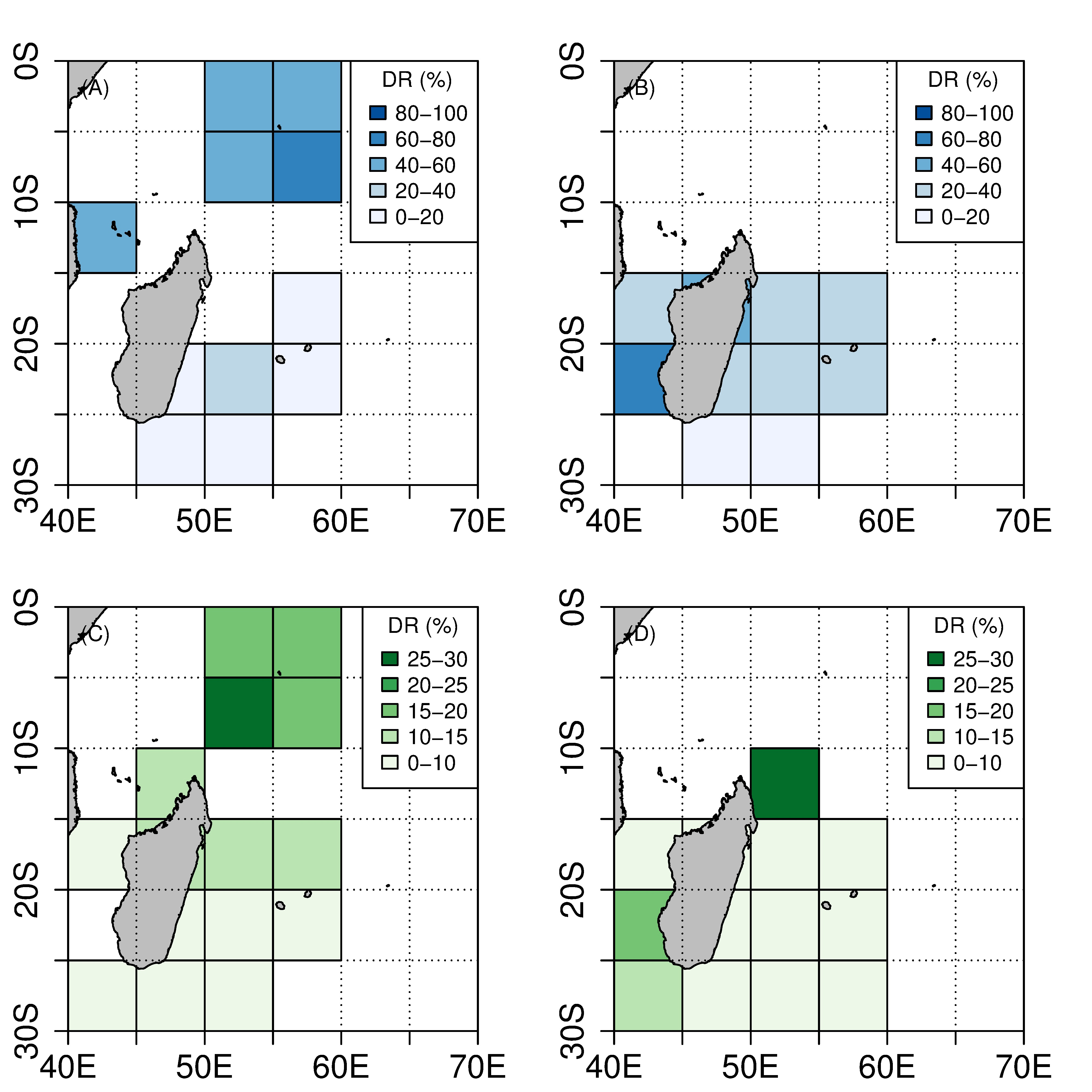

Supplement: S7 Fig — Mean DR in % (left: 2004–2010, right: 2011–2015; blue: toothed whale depredation, green: shark depredation). (TIF) [file pone.0202037.s008.tif]

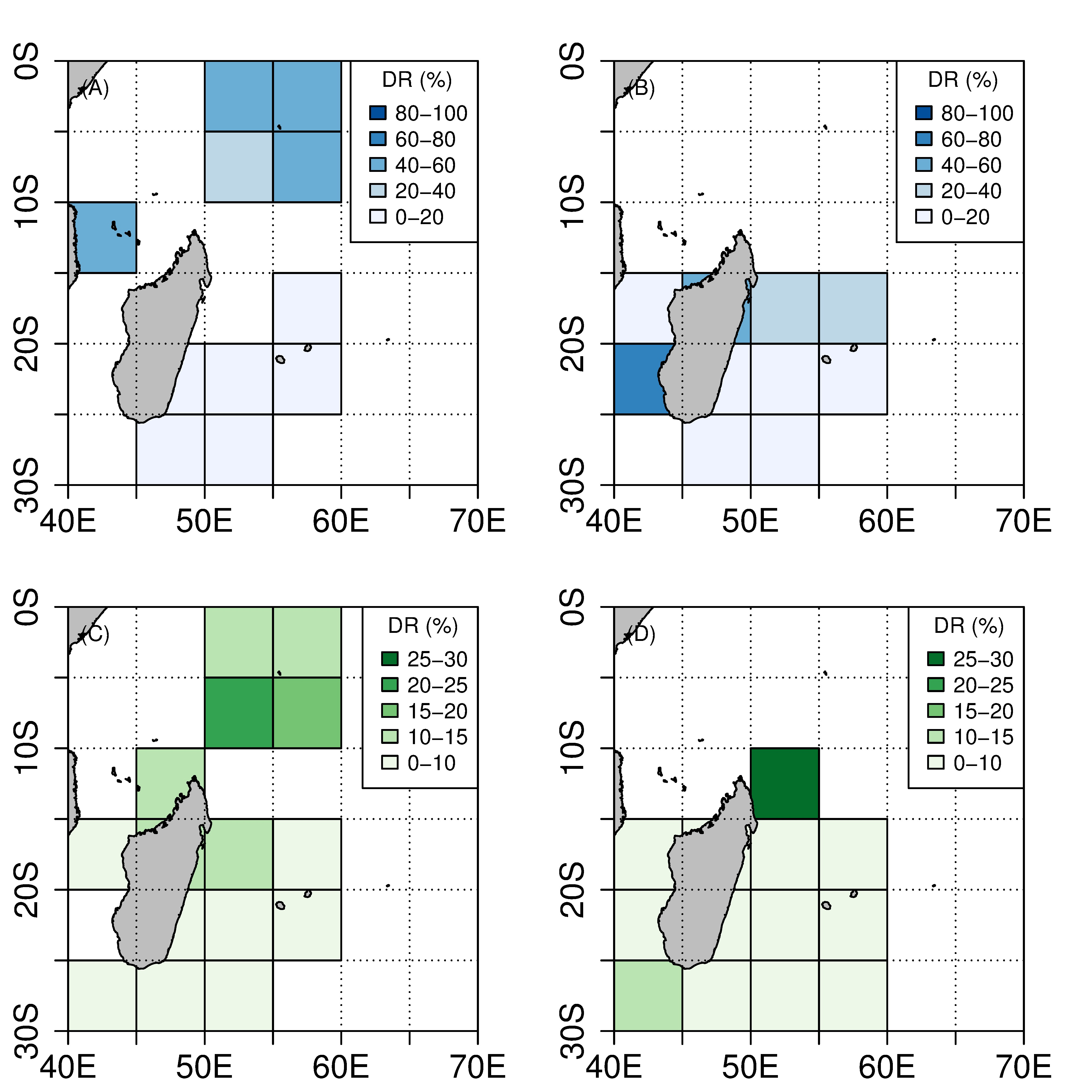

Supplement: S8 Fig — Median DR in % (left: 2004–2010, right: 2011–2015; blue: toothed whale depredation, green: shark depredation). (TIF) [file pone.0202037.s009.tif]

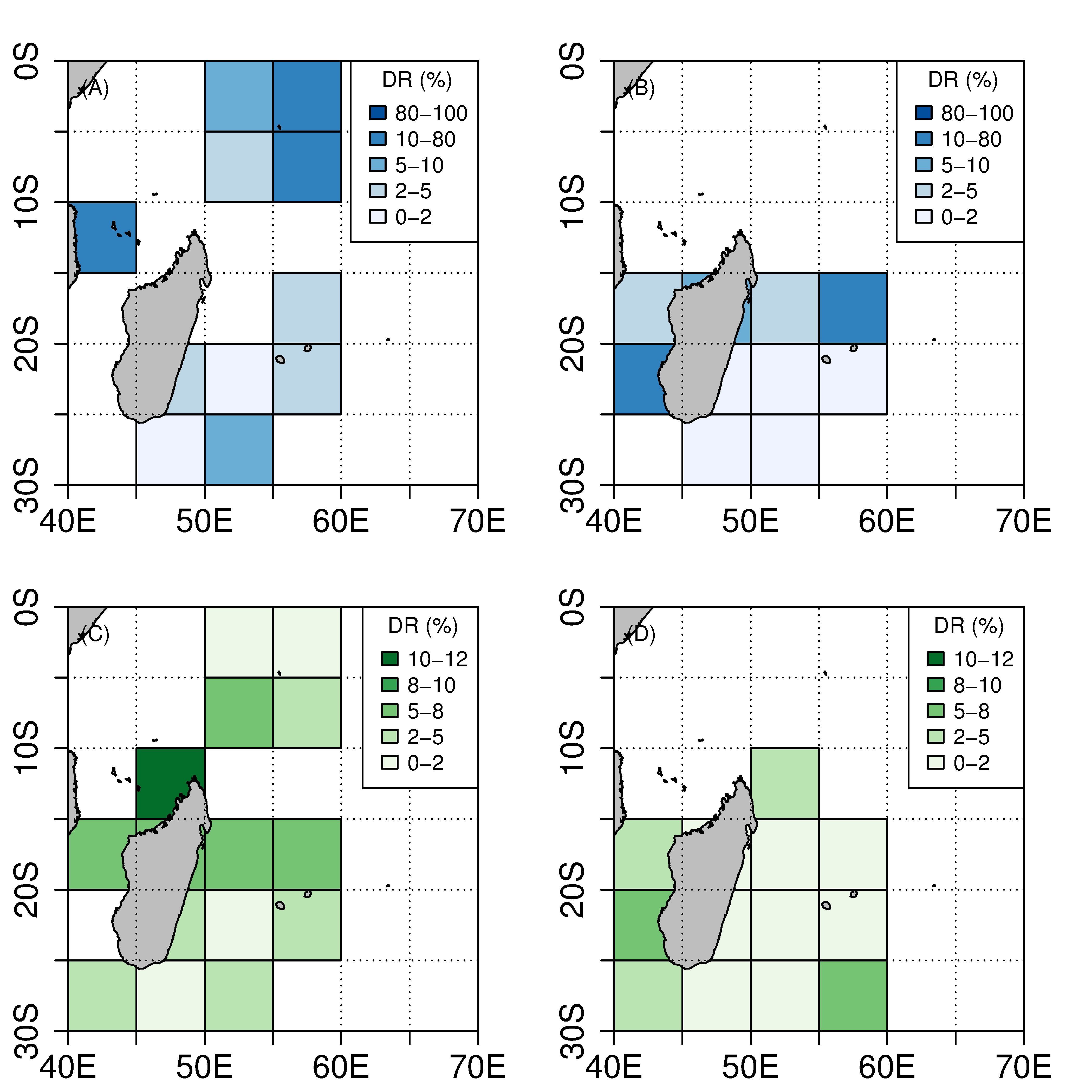

Supplement: S9 Fig — Minimum DR in % (left: 2004–2010, right: 2011–2015; blue: toothed whale depredation, green: shark depredation). (TIF) [file pone.0202037.s010.tif]

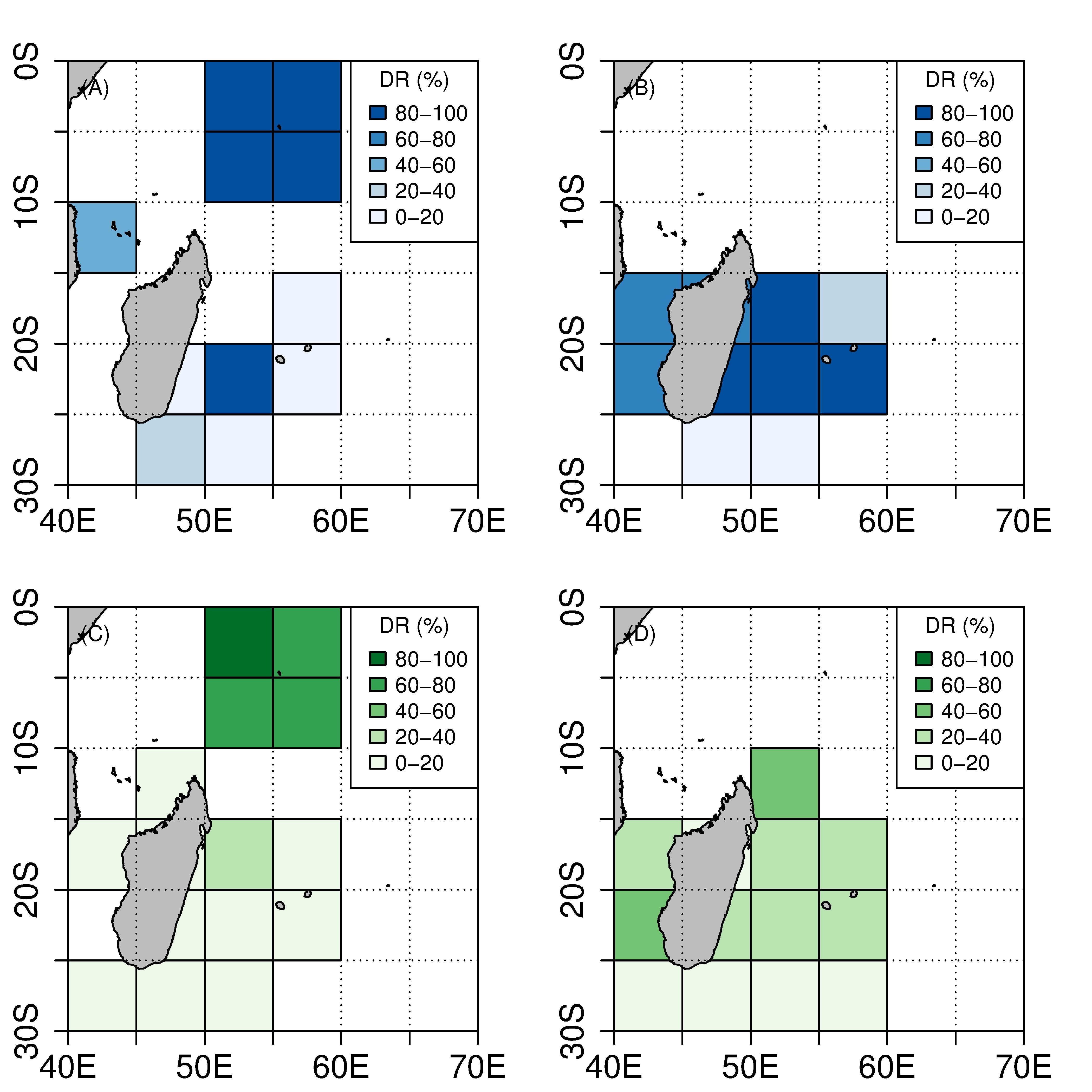

Supplement: S10 Fig — Maximum DR in % (left: 2004–2010, right: 2011–2015; blue: toothed whale depredation, green: shark depredation). (TIF) [file pone.0202037.s011.tif]

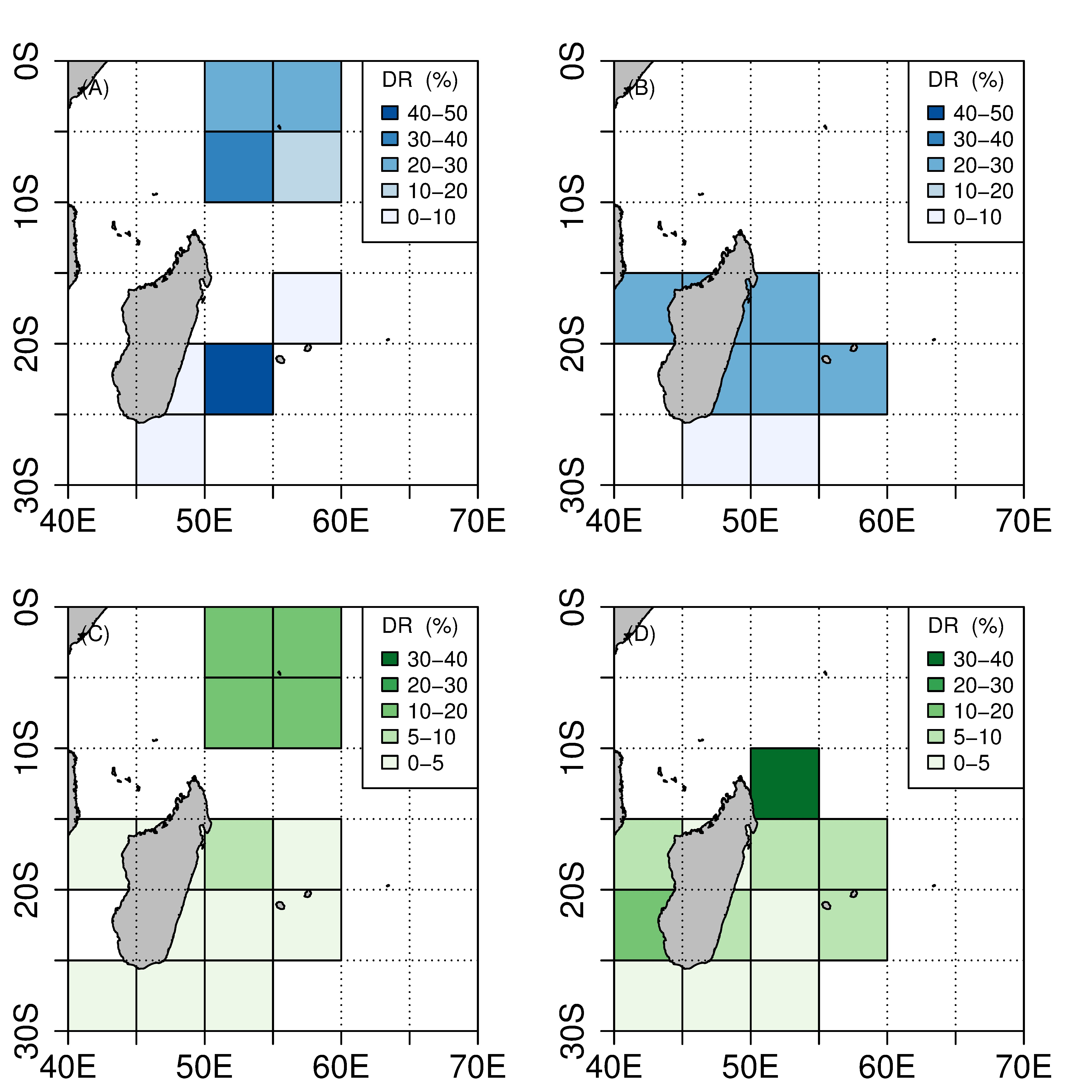

Supplement: S11 Fig — Standard deviation of DR in % (left: 2004–2010, right: 2011–2015; blue: toothed whale depredation, green: shark depredation). (TIF) [file pone.0202037.s012.tif]

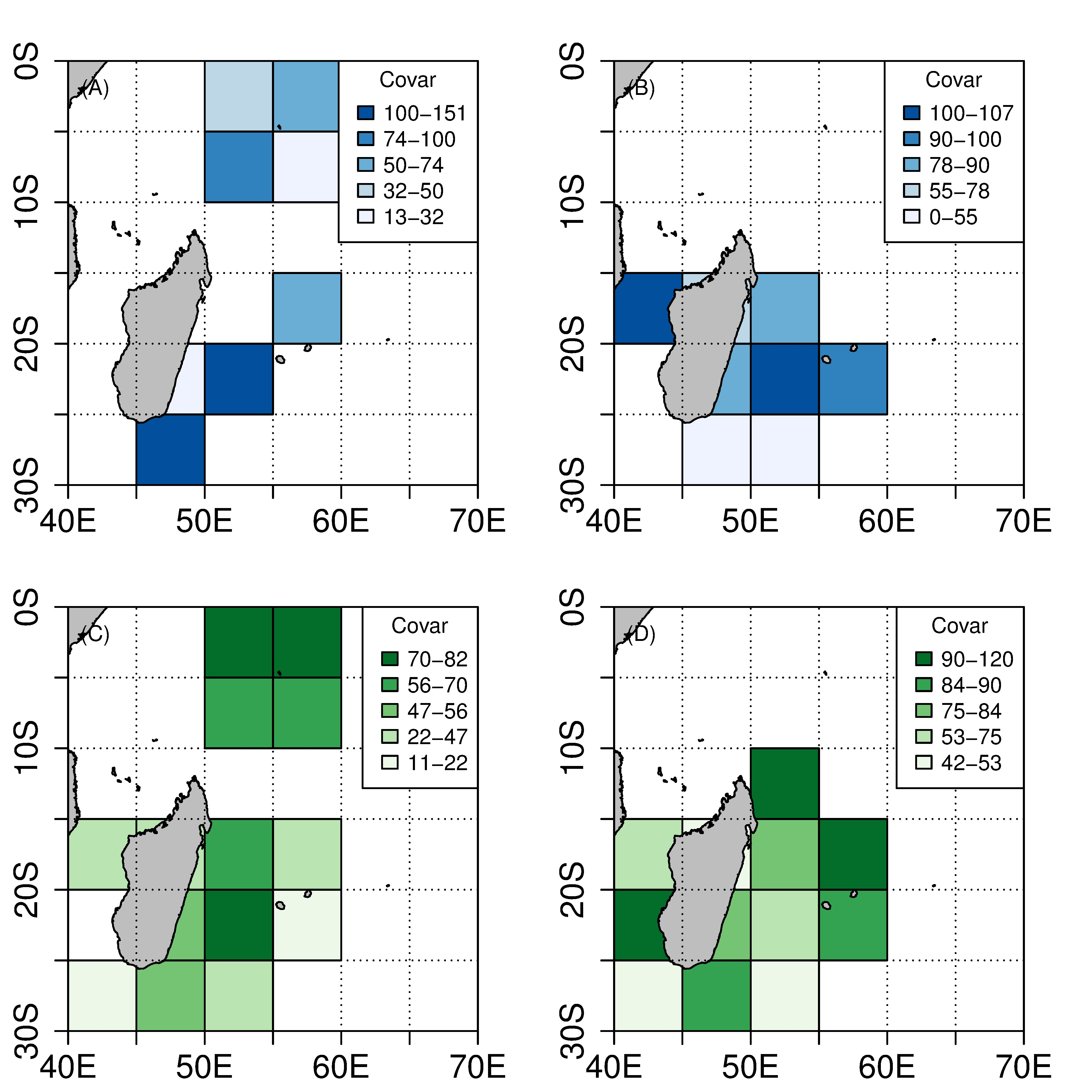

Supplement: S12 Fig — Coefficient of variation of DR in % (left: 2004–2010, right: 2011–2015; blue: toothed whale depredation, green: shark depredation). (TIF) [file pone.0202037.s013.tif]
